# Supplementary figures and images for: A new human embryonic cell type associated with activity of young transposable elements allows definition of the inner cell mass
Source: PLoS Biol. 2023 Jun 20;21(6):e3002162. doi: 10.1371/journal.pbio.3002162 (PMC10281584; doi:10.1371/journal.pbio.3002162)

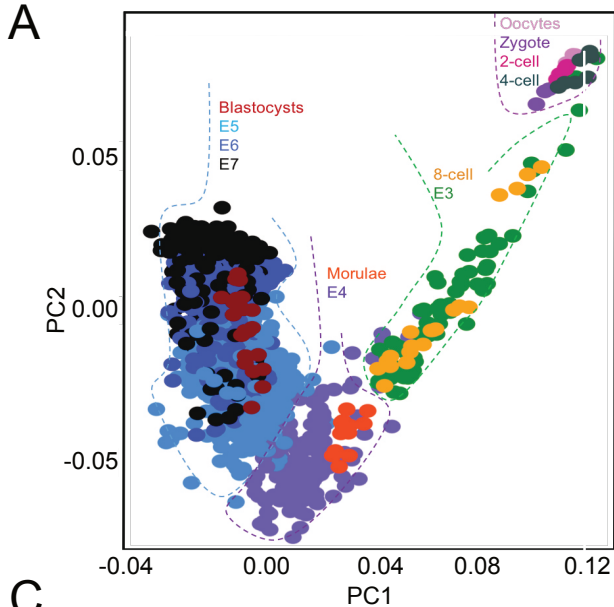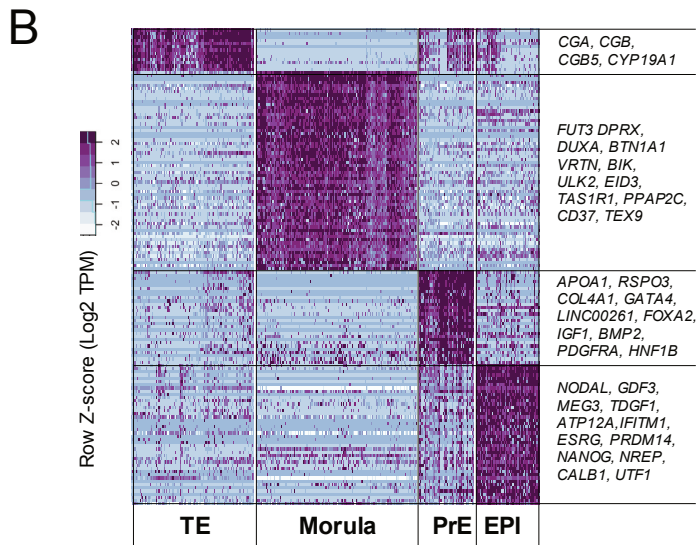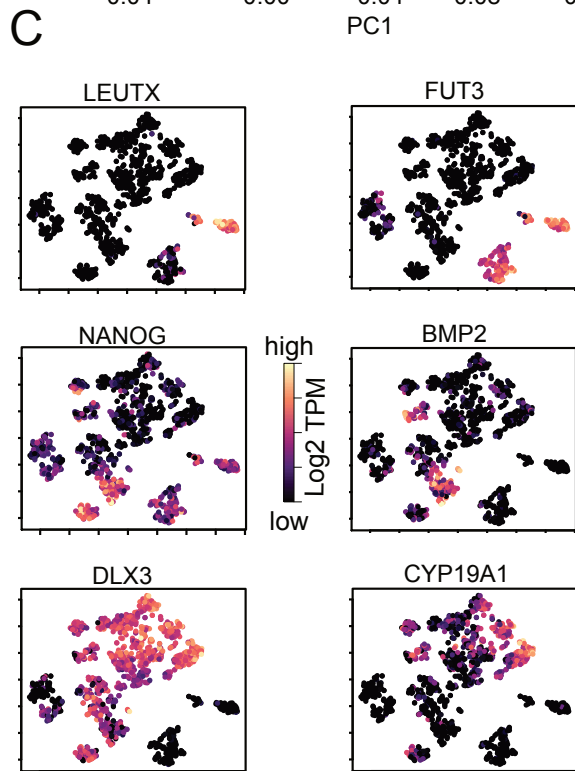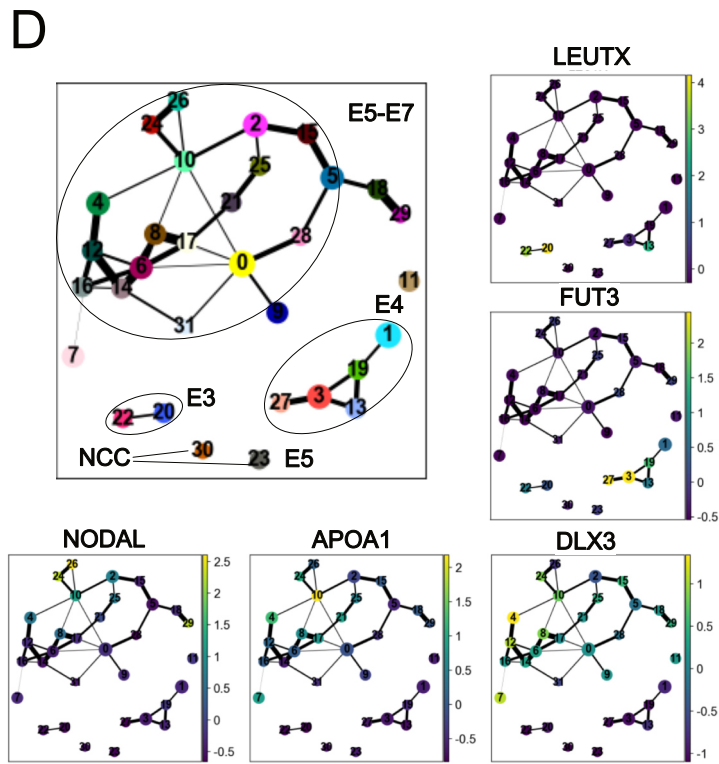

Supplement: S1 Fig — Code to generate these figures is at https://doi.org/10.5281/zenodo.7925199. (A) Tracing the human embryonic development progression from zygote to blastocyst. PCA of cross-platform 1285+114 single-cell preimplantation transcriptome [1,2] using 1,583 MVGs. Developmental stages defined as in [2,40]. Note that this figure also demonstrates that any batch effects were adequately corrected prior to the analysis: instead of clustering due to different batches, single cells cluster on the basis of their embryonic stages (E3 with 8-cell, E4 with Morula, and E5-7 with blastocysts). (B) Feature plots based on tSNE plot from Fig 1A visualising the expression of selected lineage-specific markers, e.g., LEUTX (8-cell), FUT3 (morula), NANOG (EPI), BMP2 (ICM/PrE), DLX3 (pre-TE), CYP19A1 (TE). Colour intensity gradient indicates the expression of the marker gene (black, lower; coloured, higher). Each dot represents an individual cell. (C) Heatmap displaying the scaled expression (Log2 TPM values) of discriminative gene sets (AUC cutoff ≥0.90) defining cell populations of morula (n = 171), EPI (n = 52), PrE (n = 45), and TE (n = 99) reported in Fig 1A. Heatmap colour scheme is based on Z-score distribution from −2 (light blue) to 2 (purple). Note: The majority of the markers agree with a previous study [2]. Here, we show the markers of EPI, PrE, and TE from E3–E7. (D) PAGA connectivity graph representation of raw single cell count datasets from E3 to E7 using default parameters of SCAMPY-PAGA. Coloured nodes represent subclusters of transcriptionally similar cells (threshold 0.1). The number of the cells forming a particular subcluster is reflected in the size of the circle. Numbers indicate cluster ID. Similarity between subclusters is indicated by connecting lines (thickness denotes the statistical measure of connectivity between clusters). Subclusters (circled) can form higher order categories that correspond to developmental stages of 8-cell, morula, and the blastocyst at E3, E [file pbio.3002162.s001.pdf]

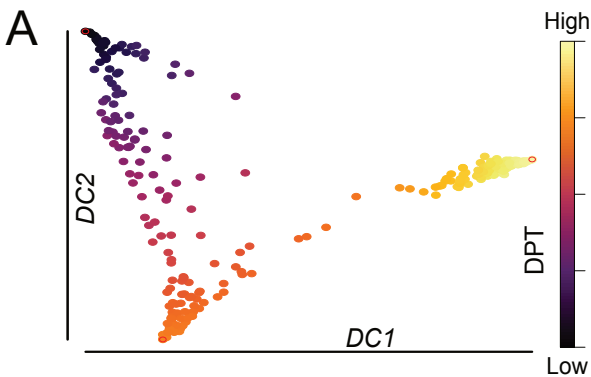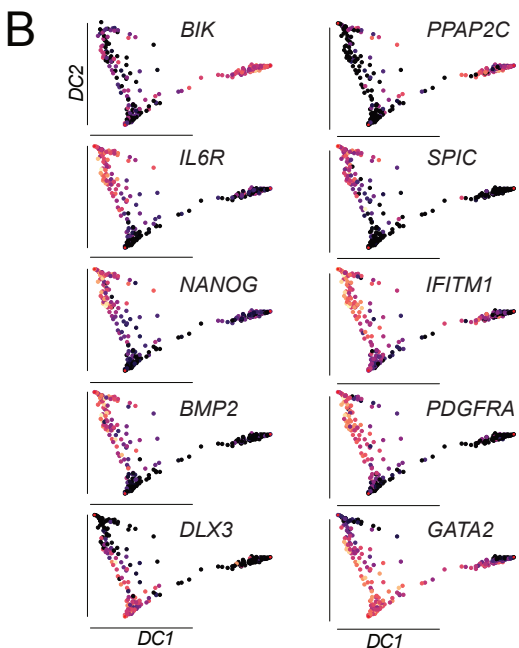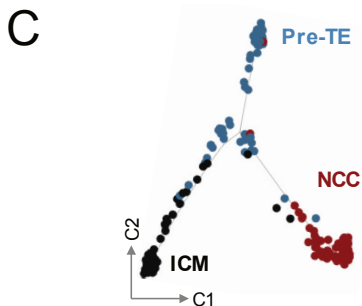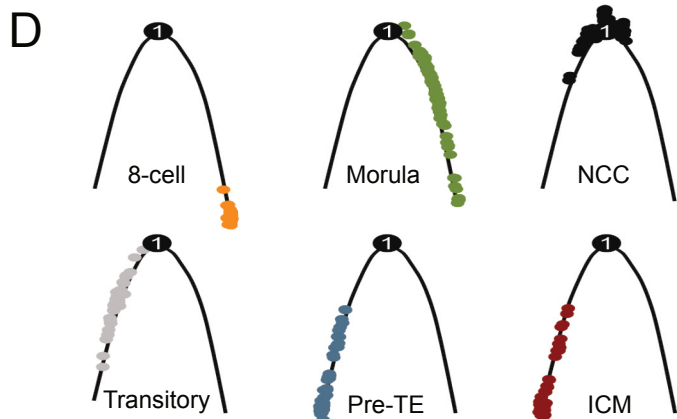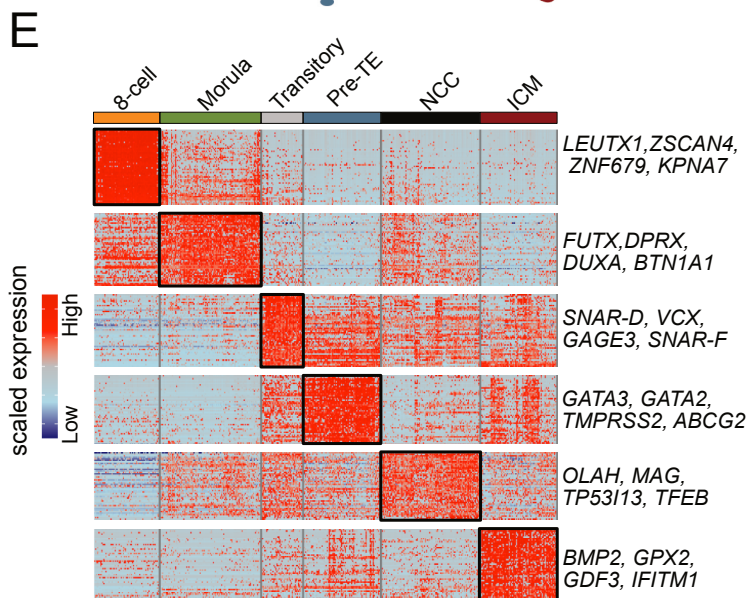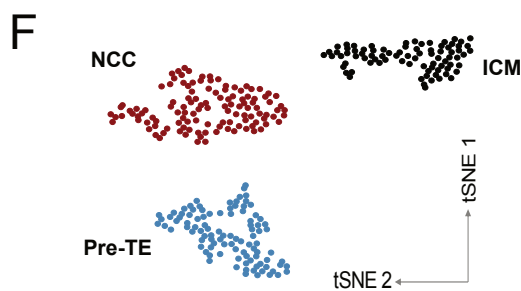

Supplement: S2 Fig — Code to generate these figures is at doi.org/10.5281/zenodo.7925199. (A) DPT plot between Diffusion Component 1 and 2 (DC1 and DC2) illustrating 3 major states of E5 along pseudotemporal ordering. Bottom: pre-TE. top: ICM. Middle right: NCCs and cells are under progression. Cells coloured dark and golden yellow are representing higher and lower diffusion values. (B) The series of feature plots show the expression dynamics of individual genes in E5 blastocyst plotted on the DPT (A). Cells coloured dark and golden-yellow are representing higher and lower expression of respective genes, respectively. ICM (e.g., IL6R and SPIC) is characterised by the progressing cells enriched in EPI (e.g., NANOG and IFITM1) and PrE (e.g., BMP2 and PDGFRA). pre-TE population is identified by marker gene expression (e.g., DLX3 and GATA2). Note that NCCs do not express any lineage markers, but are marked by BIK and PPAP2C expression. (C) Pseudotime trajectory showing the ordering of E5 cells. Monocle2 visualisation of pre-TE, ICM, and NCC trajectories using the DDRTree algorithm. The top 2,000 DEGs are projected into a 2D space (right panel). (D) Monocle2 single cell trajectory analysis and ordered cells along an artificial temporal continuum using the top 1,000 DEGs across the data frame of E3–E5 cells. The transcriptome from each single cell represents a pseudotime point along an artificial time vector that denotes the progression from 8-cell to blastocyst via morula. Note: The artificial time point progression agrees with the biological one. NCCs deviate on the trajectory. We show the trajectory in 6 facets, one for each cluster identified previously (Fig 1A). Colour codes as in (C). (E) Heatmap visualisation of scaled expression [log TPM (transcripts per million)] values of distinctive set of 1,000 genes (AUC cutoff >0.90) for each trajectory shown on (C). (AUC cutoff >0.90). Colour scheme is based on Z-score distribution from −2.5 to 2.5. Top margin colour bars highlight representati [file pbio.3002162.s002.pdf]

A

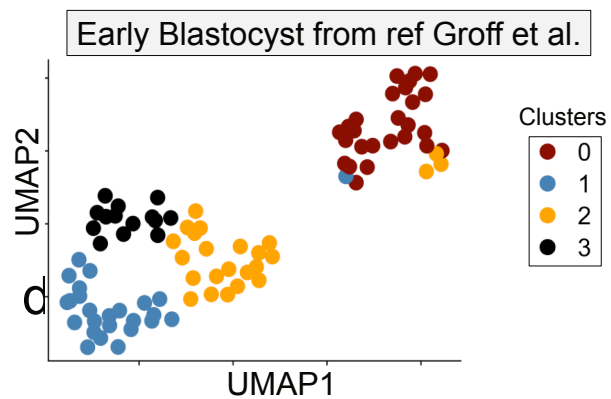

B

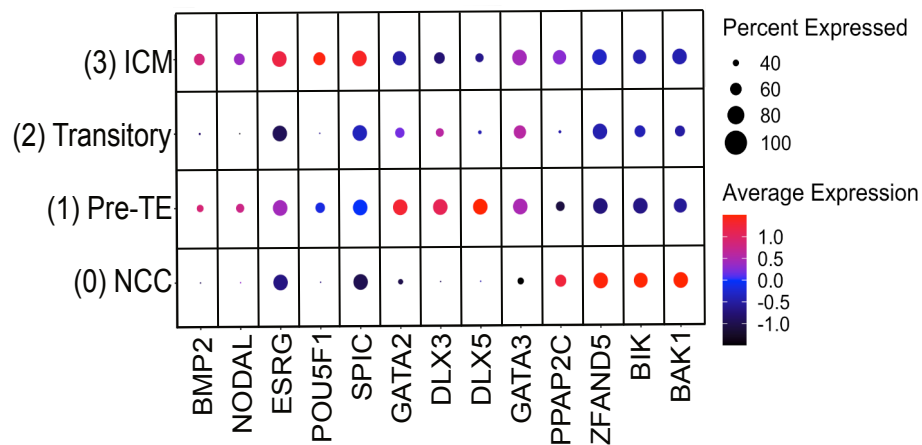

C

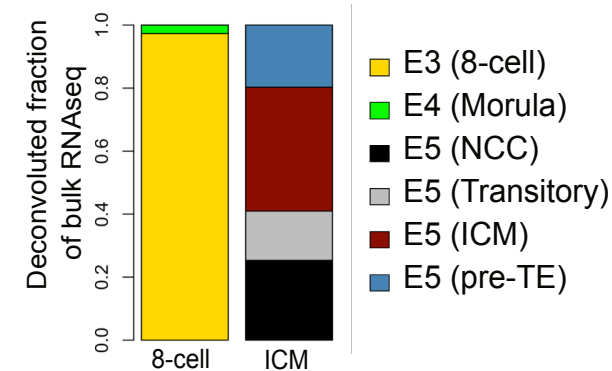

D

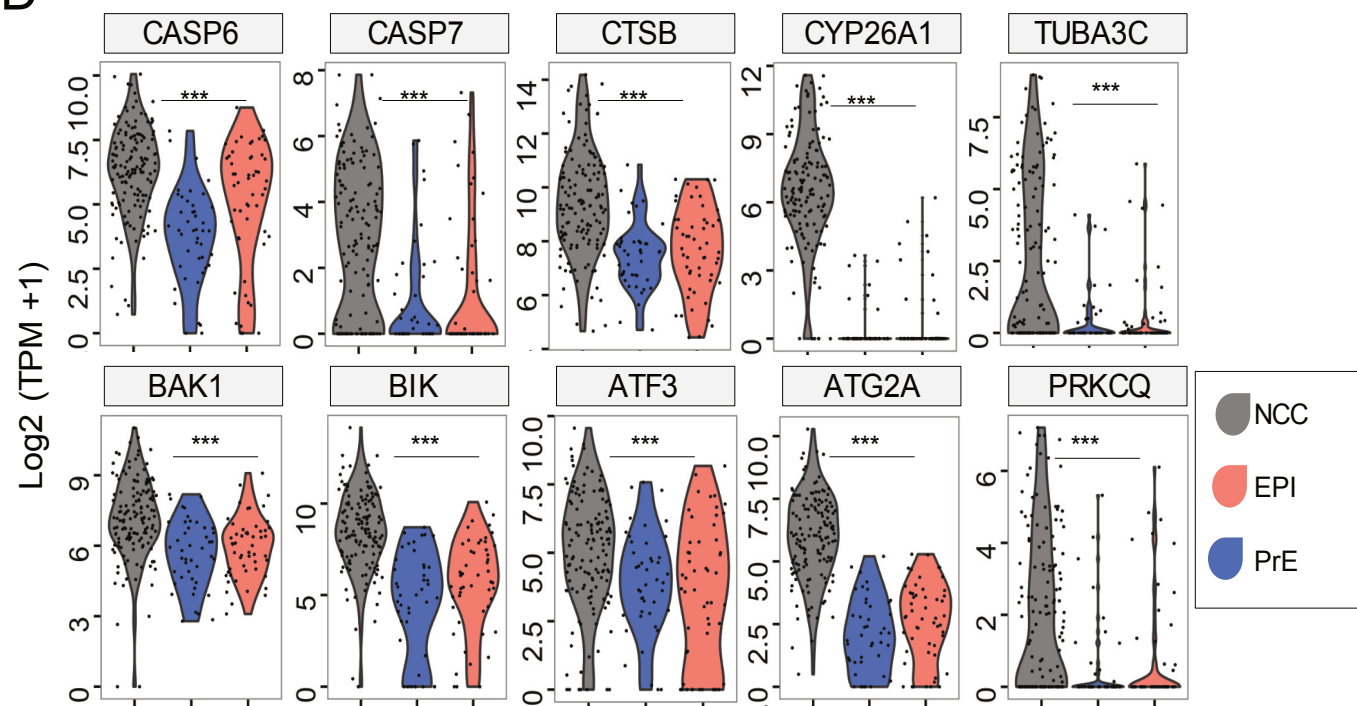

E

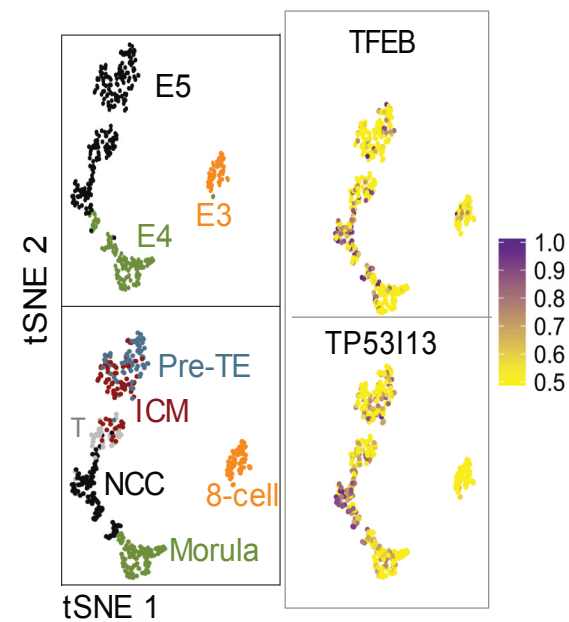

Supplement: S3 Fig — Code to generate these figures is at doi.org/10.5281/zenodo.7925199. (A) UMAP clustering of E5 cells from an independent study [17] using approximately 1,000 MVGs reveals 4 distinct cell populations of pre-TE, ICM, Transitory, and NCCs (see the markers in (B)). Each dot represents a single cell. (B) RNA transcript intensity and density of differentially expressed markers of ICM, Transitory, pre-TE, and NCC clusters across the cell types obtained by the UMAP clustering (A). The dot colour scales from blue to red, corresponding to lower and higher expression, respectively. The size of the dot is directly proportional to the percentage of cells expressing the markers in a given cell type. (C) Reference based deconvolutions on bulk RNAseq (8-cell and ICM, GSE101571) and scRNAseq datasets using data from E3–E5 lineages. The stacked barplot shows the identified E3–E4 transcriptomes in the 8-cell bulk RNAseq, whereas the ICM-E5 sample has the identifiable lineages of Transitory, ICM, pre-TE, and NCC. We employ the single cell marker evidence to classify proportions of cell types. As these markers of NCC are apoptotic markers, we conclude that, in agreement with visualisations and scRNA data, approximately 20% of cells are NCC type. (D) Multiple violin plots visualise the density and distribution of expression (Log2 TPM values) of selected genes that are up-regulated in human NCC vs. EPI/PrE. The depicted genes (top candidates) are involved in regulating apoptotic pathways (KEGG: hsa04210, Gene Ontology GO:008219, GO:0012501, and GO:0006915) (Wilcoxon test, p-value < 7.135 × 10^−6^). (E) Feature plots based on tSNE plot (also shown on S5F Fig) demonstrating lineage-specific expression of apoptotic genes responding to DNA damage, e.g., TFEB [19] or TP53I13, the latter associated with telomere maintenance and genome stability [92]. Dots in yellow denote lower, whereas purple denote higher level of gene expression in a given single cell. (PDF) [file pbio.3002162.s003.pdf]

A

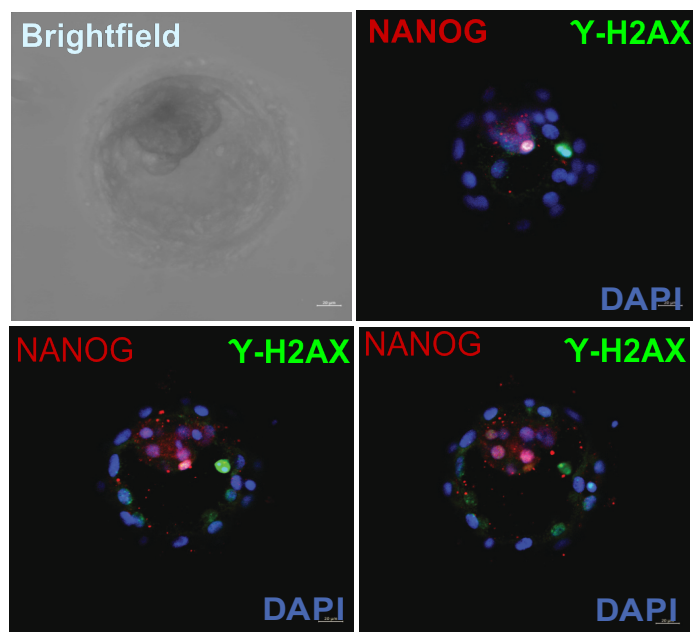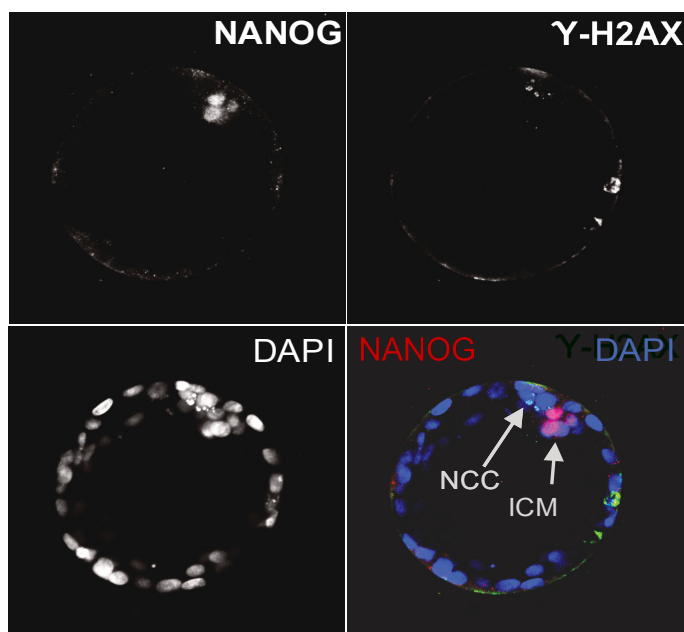

B

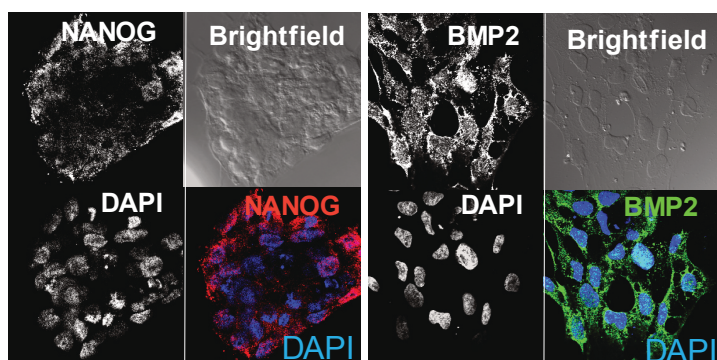

C

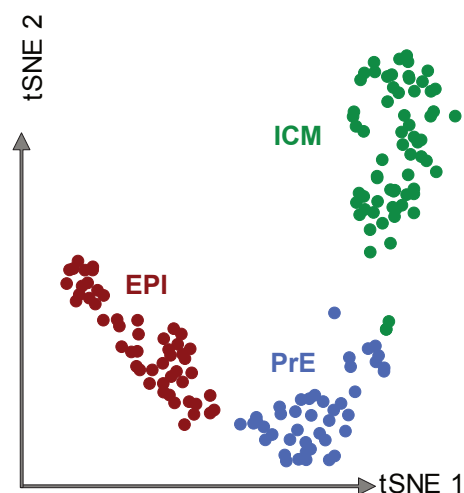

D

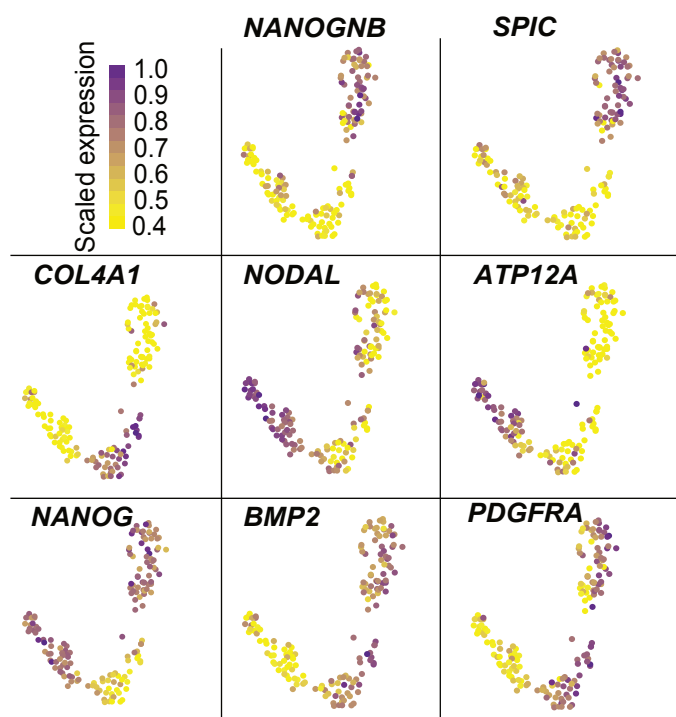

E

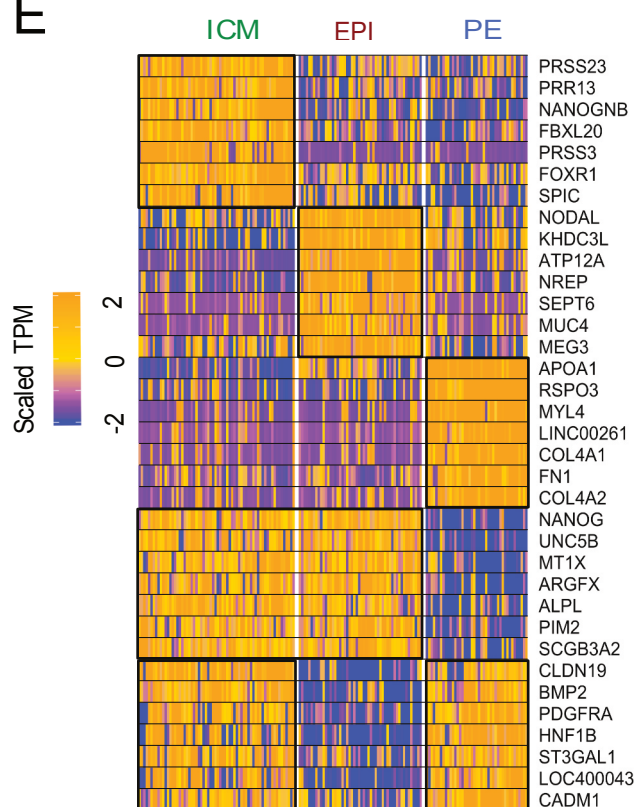

Supplement: S4 Fig — Code to generate these figures is at doi.org/10.5281/zenodo.7925199. (A) Representative confocal immunofluorescent images of human E5 blastocysts co-stained against NANOG (red), γ-H2AX (green), and DAPI (blue); brightfield, black-and-white panels. Cells in the blastocyst are stained either and exclusively with NANOG, representing the compacting cells of the ICM, or with γ-H2AX, representing damaged/dying cells. Note that the γ-H2AX+ cells with disintegrating nucleus are distinct form the committed, pre-TE cells. Images of 3 different embryos are shown. Magnification is 40×. Venn diagram shows the numerical analysis of the immunofluorescent co-staining performed on the 3 independent embryos. See also Fig 2A. (B) Optimisation of immunostaining using NANOG (red) and BMP2 (green) antibodies in hESC_H9 cells. Magnification is 63×. (C) tSNE biplot visualises the human ICM (n = 71), PrE (n = 45), and EPI (n = 52) clusters using the most variable genes (n = 532) (see S3 Table for the full list of the markers). ICM is E5, EPI and PrE are E6-7. (D) Multiple feature plots based on tSNE plot from (C) displaying unsupervised identification of expression markers of ICM (NANOGBB, SPIC), ICM/EPI (NANOG, SCGB3A2), ICM/PrE (BMP2, PDGFRA), EPI (NODAL, ATP12A), and PrE (COL4A1, APOA1) (see S3 Table for the full list of the markers). Dots in yellow/purple denote lower/higher expression in a given single cell, respectively. (E) Heatmap showing scaled expression (log TPM values) of distinctive marker gene sets defining EPI, PrE, and ICM. Genes specific to ICM include NANOGNB, PRSS3, and SPIC. Note: the progenitor markers that are homogeneously expressed in ICM, but also in EPI (e.g., NANOG, MT1X) or PrE (e.g., BMP2, PDGFRA). Note: Underlined genes are specific to human when compared with macaque. Colour scheme is based on Z-score distribution, from –2.5 (gold) to 2.5 (purple). (PDF) [file pbio.3002162.s004.pdf]

A

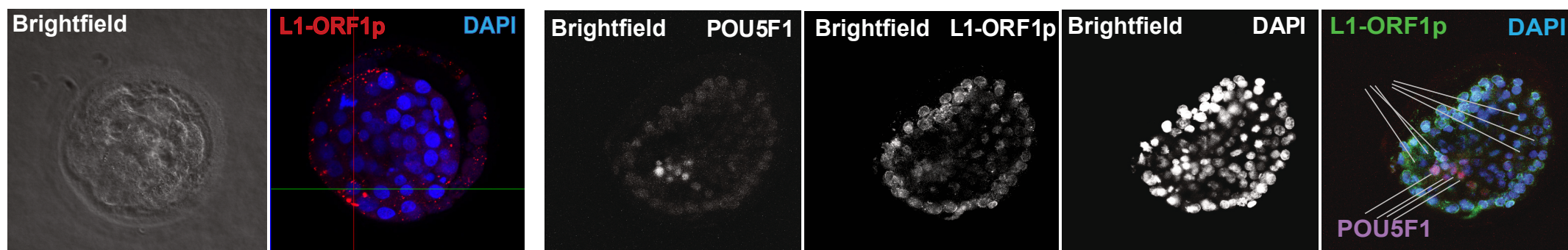

B

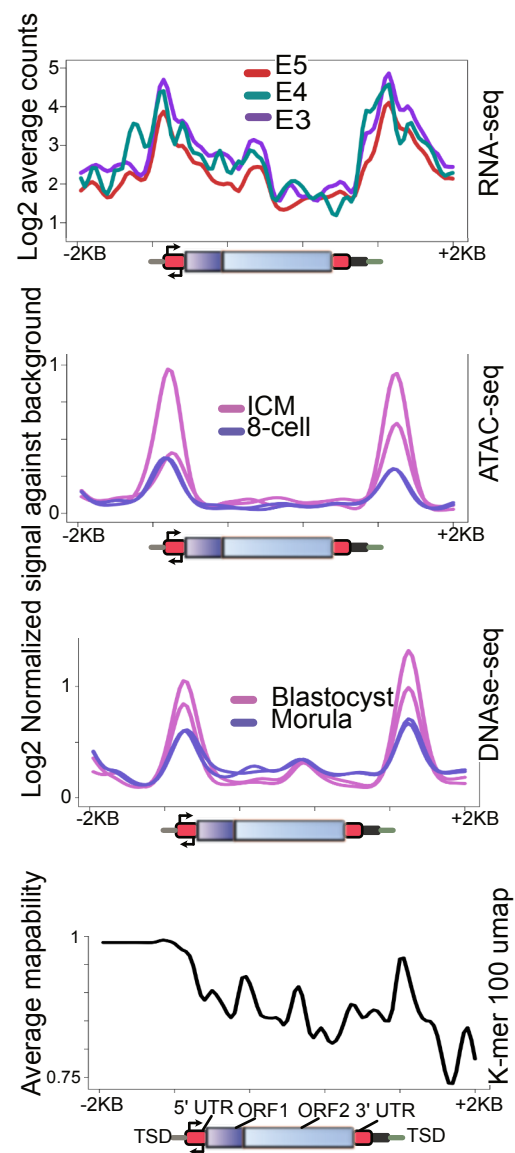

C

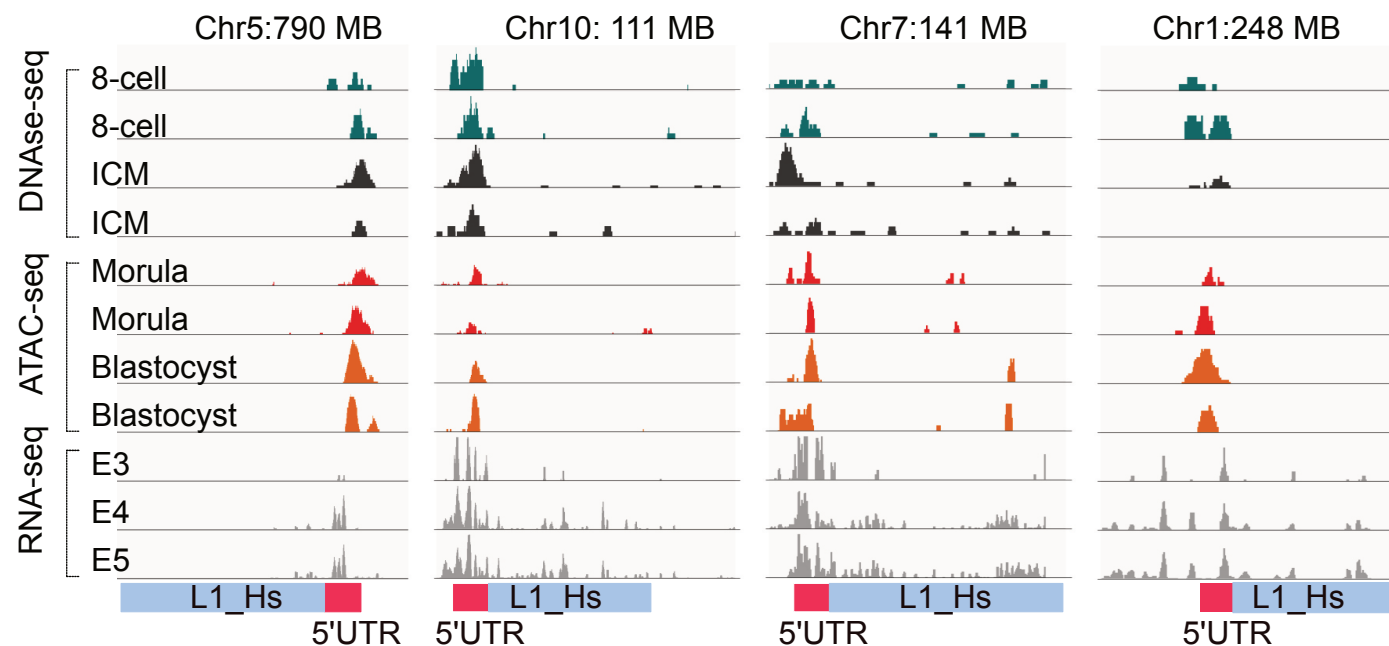

D

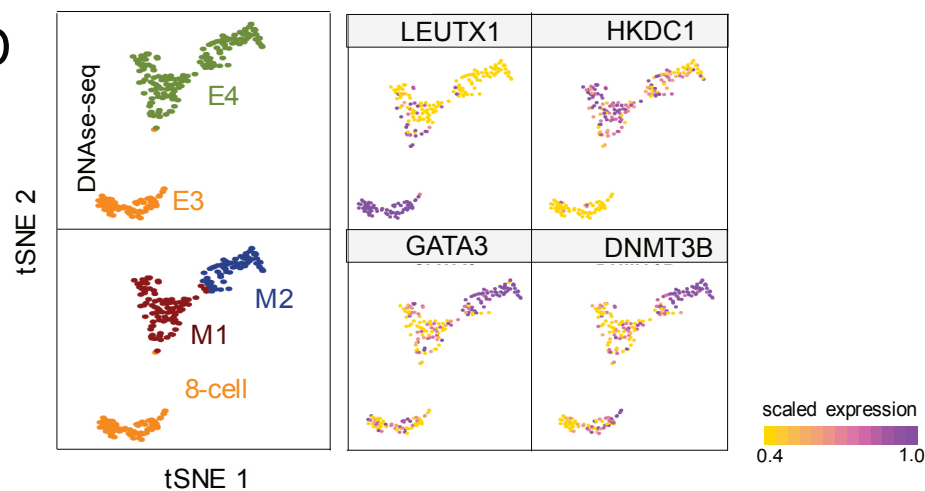

Supplement: S5 Fig — Code to generate these figures is at doi.org/10.5281/zenodo.7925199. (A) A representative confocal immunofluorescent image of a human E5 blastocyst co-stained against L1-ORF1p (left 2 panels) (cytoplasmic, red) and DAPI (nuclear, blue; brightfield, black-and-white panel). Note: the scattered L1-ORF1p signal also in TE. Magnification is 40×. See also S1 Movie. Representative confocal immunofluorescent images of a human E5late blastocyst (right 4 panels) (brightfield, black-and-white panels) co-stained against POU5F1/OCT4 (nuclear, purple), L1-ORF1p (cytoplasmic, green), and DAPI (nuclear, blue). Note: The antagonistic expression of POU5F1/OCT4 and L1-ORF1p: POU5F1 expression stains the compacting cells, where L1-ORF1p expression is not detectable. L1-ORF1p+ cells are also detectable in TE. Magnification is 63×. (B) Regulation the expression of “hot” L1 elements in blastomeres and ICM. RNA-seq coverage shows the mapable reads; ATAC-seq/DNAse-seq coverage plots show the signals around the transcription start sites (TSSs) located at the left boundaries of “hot” L1 loci. X-axis, upstream 2 KB and downstream 8 KB regions from the left boundaries divided into 100 bins, each comprising 100 bps; Y-axis, normalised ATAC-seq signal (per million per 100 bp bin). Samples: Replicates (n = 2) of human 8-cell stage/morula embryo, human bulk-ICM. (C) Activity of 4 of the 6 “ultra-hot” L1 elements [10] in early human development (data source as in (B)). (D) tSNE unbiased clustering of E3–E4 cells using approximately 500 MVGs reveals 3 distinct cell populations (upper left panel). The 3 groups of cells are identified as 8 cells stage and 2 distinct populations of E4 embryo Morula 1 (M1) and Morula 2 (M2) (lower left panel). Each dot represents a single cell. On the right panels, we show feature plots based on tSNE plot demonstrating lineage-specific expression of LEUTX (8-cell marker [1,2,93]), HKDC1 (M1 marker, this study), GATA3 (M2 marker, this study, but pre-TE marker in [6,40,93] [file pbio.3002162.s005.pdf]

A

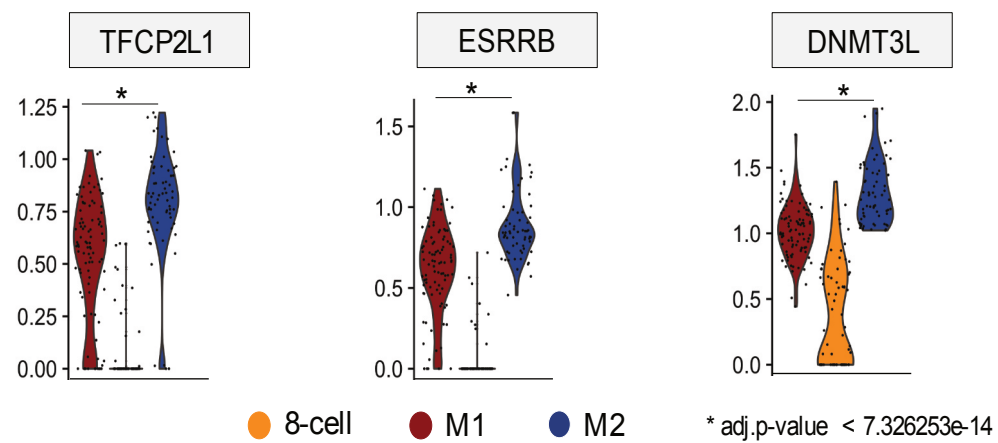

B

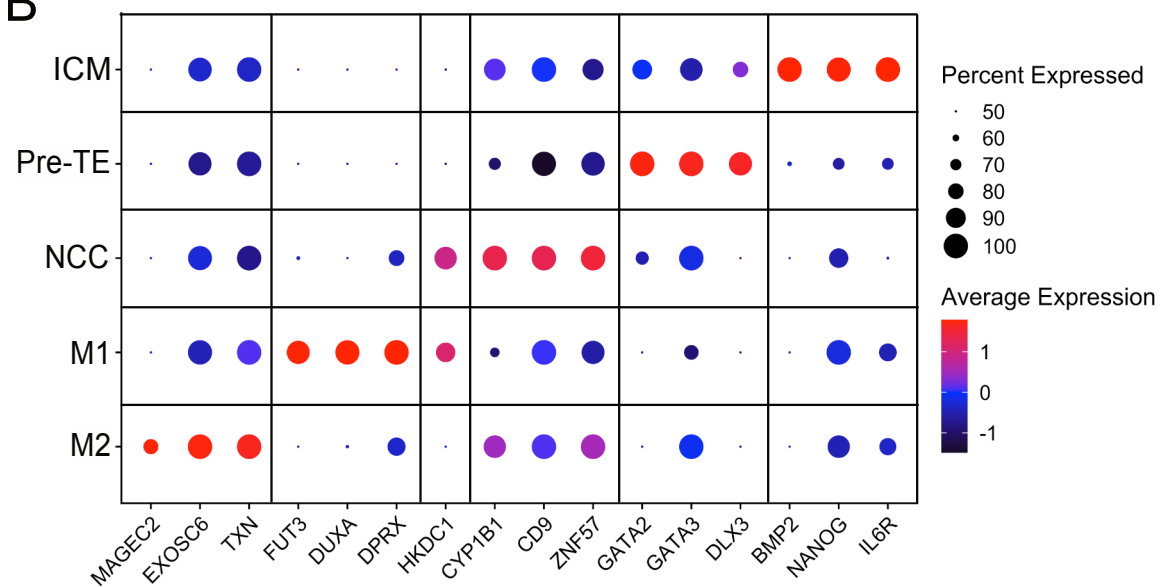

C

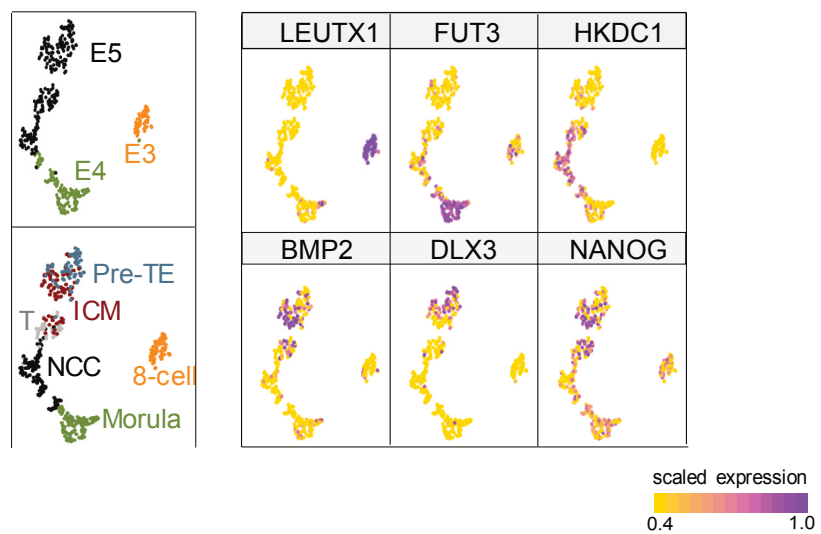

D

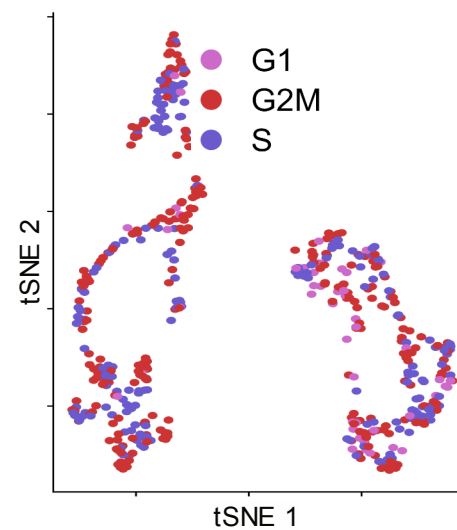

E

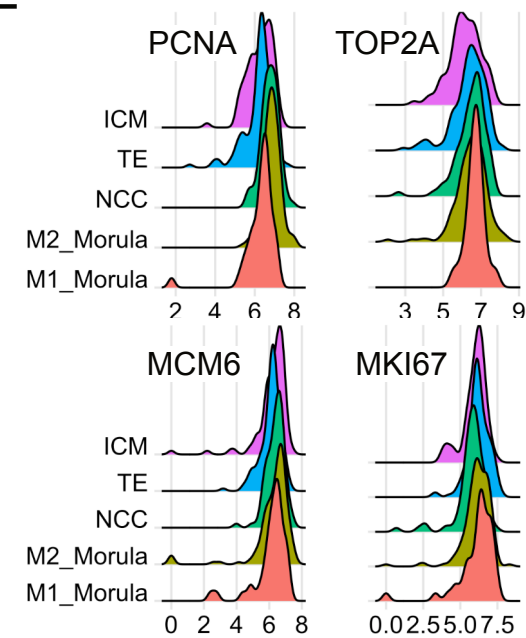

Supplement: S6 Fig — Code to generate these figures is at doi.org/10.5281/zenodo.7925199. (A) Violin plots showing the expression distribution of TFCP2L1, ESRRB, and DNMT3L (naïve stem cell culture markers [94–96]) in 8-cell and morula, M1 and M2 stages. Adjusted p-values are obtained by Benjamini–Hochberg (BH) correction. Each point represents a single cell. (B) Dot plots of differentially expressed genes marking each cluster of cells shown in S5F Fig. The dot colour scales from blue to red, corresponding to lower and higher expression, respectively. The size of the dot is directly proportional to the percentage of cells expressing the markers in a given cell type. (C) tSNE clustering of E3–E5 cells from approximately 1,000 MVGs obtained using default parameters of “Seurat” package (upper panel) dissects 5 distinct groups of cells (upper left panel). The analysis of E5 blastocyst identifies pre-TE, ICM, and NCC. Transitory cells (T) are observed between ICM and NCC. Each dot represents an individual cell plotted on first 2 tSNE (lower left panel). On the right panels, we show feature plots based on the tSNE plot demonstrating lineage-specific expression of LEUTX (8-cell marker [1,2,93]), FUT3 (morula marker [1,2]), HKDC1 [97] (morula M1 and NCC marker, this study), NANOG-BMP2 (ICM marker, this study), and DLX3 (pre-TE marker [2]). Dots in purple denote higher level of gene expression in a given single cell. (D) Cell-cycle scoring of E4 and E5 single cells with their cell-cycle characteristics. (E) Cell-cycle scoring of E4 and E5 single cells with their cell-cycle characteristics, as mainly inferred by the expression of key cell-cycle genes (ridge plots). (PDF) [file pbio.3002162.s006.pdf]

A

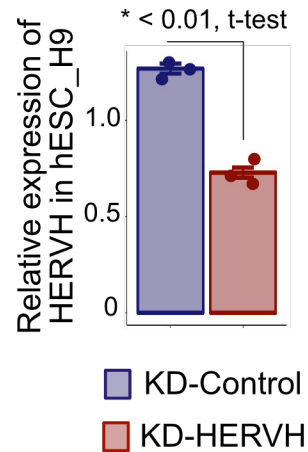

B

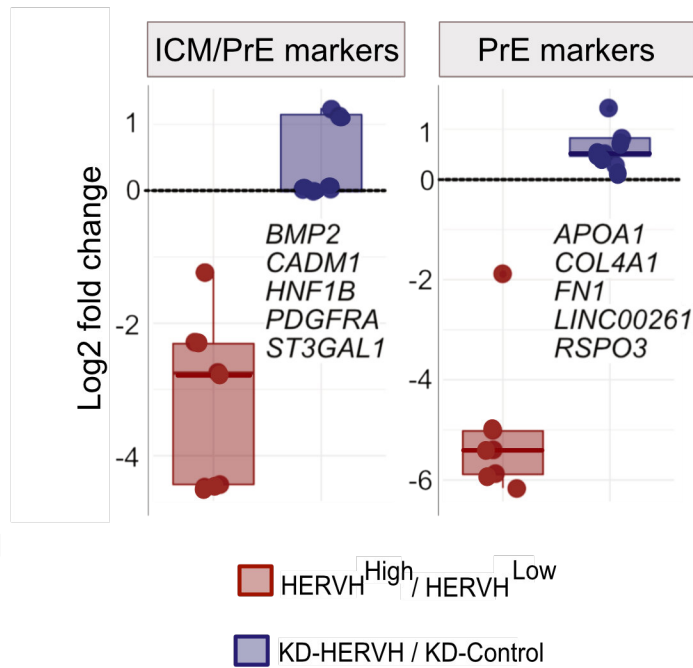

C

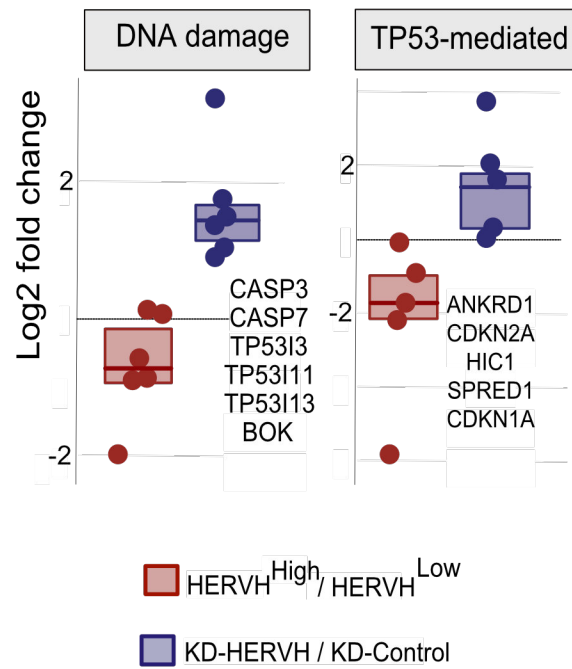

D

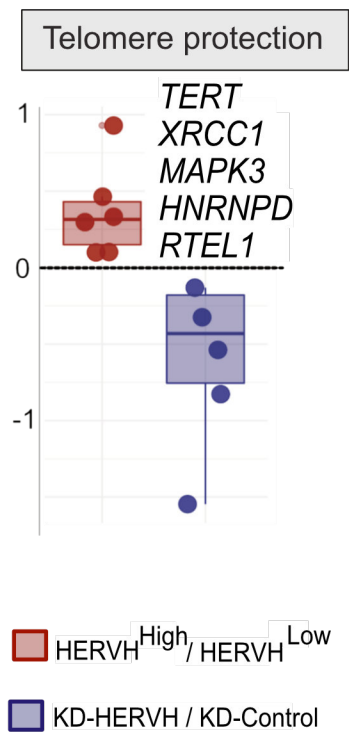

E

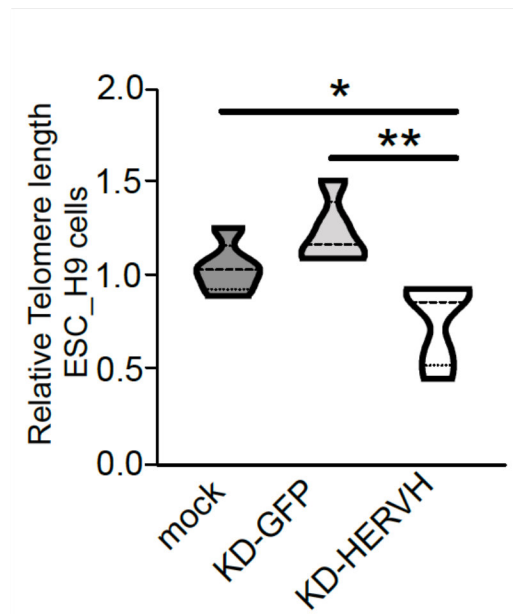

F

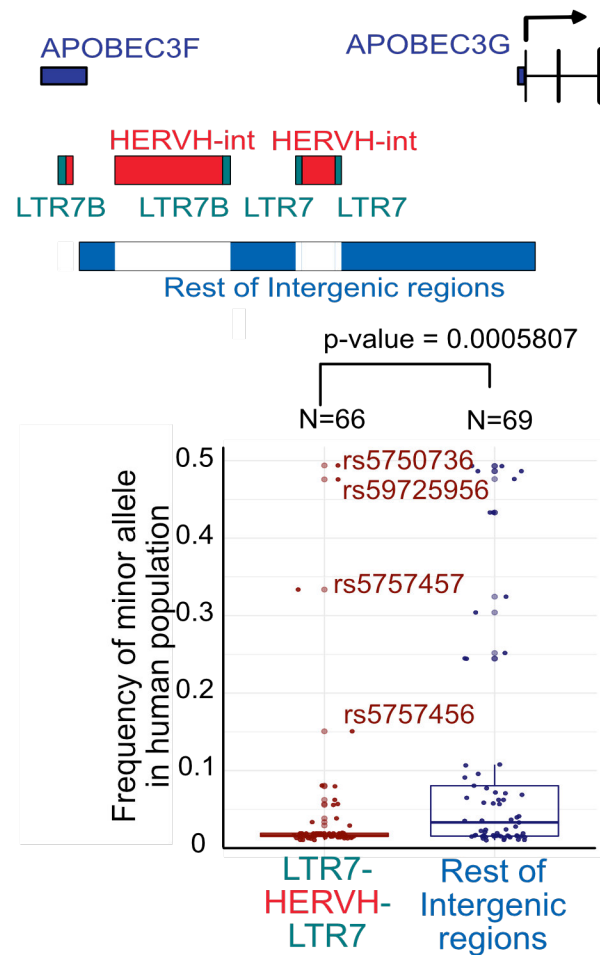

Supplement: S7 Fig — Code to generate these figures is at doi.org/10.5281/zenodo.7925199. (A) Validation of HERVH depletion (KD-HERVH) using qPCR in hESC_H9s in 3 biological replicates. KD-Control (depleted GFP). (B) The effect of HERVH expression on lineage specification. Multiple jittered boxplots display the differential gene expression (DEG, Log2-fold change) of various blastocyst (ICM/PrE; PrE) lineage markers (red, HEVHHigh vs. HERVHLow cells; blue KD-HERVH vs. KD-GFP (control) in hESC_H1s). We show the top 5 markers. Solid dots represent the differential expression values of the individual genes in the boxplots. (C) The effect of HERVH expression on genome stability. Multiple jittered boxplots display the differentially expressed genes (DEG, Log2-fold change) of various genes of GO: DNA damage responsive genes to induce apoptosis and TP53-mediated genes responsive to DNA damage (dark red, HERVHHigh vs. HERVHLow cells; blue KD-HERVH vs. KD-GFP in hESC_H1s (control)). We show the top 5 to 6 markers. (D) The effect of HERVH expression on telomere protection. Jittered boxplot displays the DEGs (Log2-fold change) of GO category: Positive regulation of telomere maintenance (red, HERVHHigh vs. HERVHLow cells; blue KD-HERVH /hESCs_H1 vs. KD-GFP/hESC_H1s (control)). We show the top 5 markers. Solid dots represent the differential expression values of the individual genes in the boxplots. (E) Knocking down HERVH shortens the telomeres in hESC_H1. Telomere length was quantified from genomic DNA using qPCR 48 h after KD-HERVH, KD-Control (scrambled) and mock transfection. KD-HERVH significantly reduced telomere length (P = 0.0317 and P = 0.0079 compared with mock and scramble control, respectively; Mann–Whitney U-test). Data are presented as violin plots with telomere length +/− 0.16 kb per chromosome end and normalised to distribution in 5 biological repeats. (F) The LTR7/HERVH-int is fixed in the human population. (Upper panel) The structure of the genomic region upstream of the APOBEC3G l [file pbio.3002162.s007.pdf]

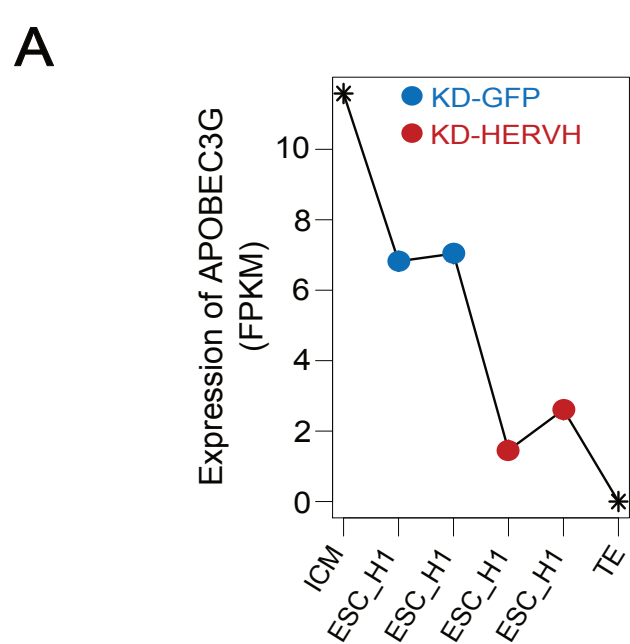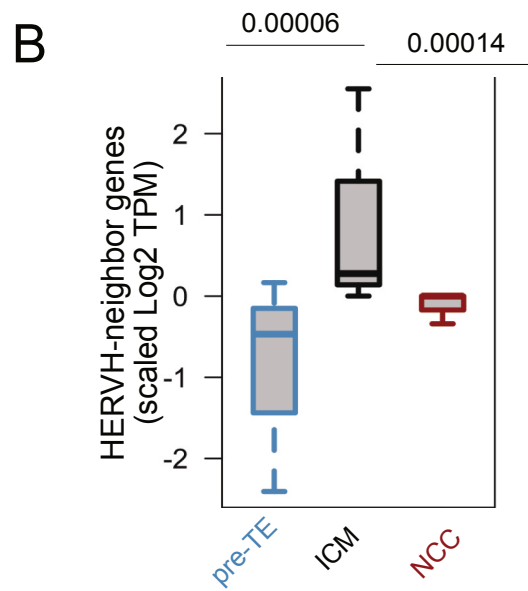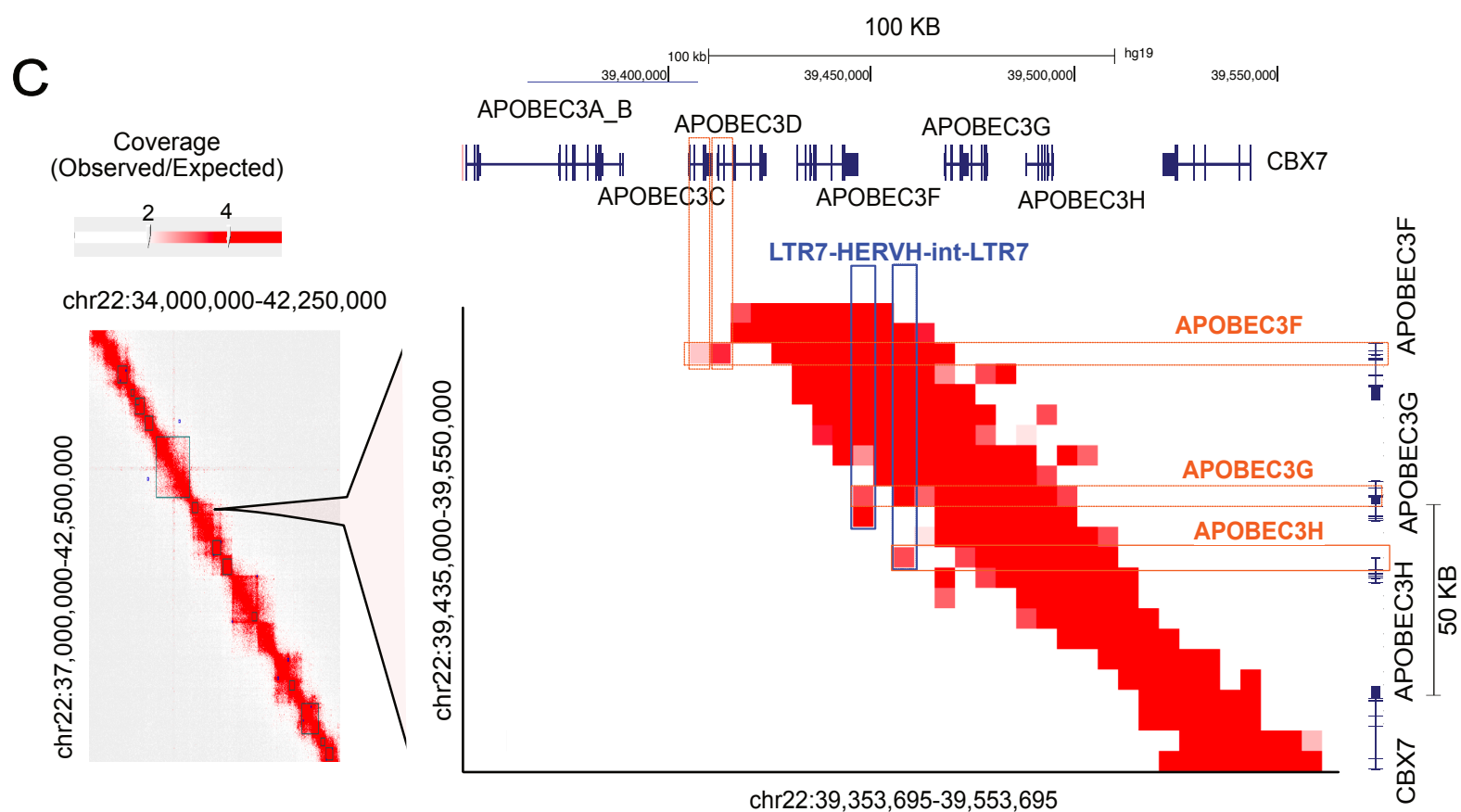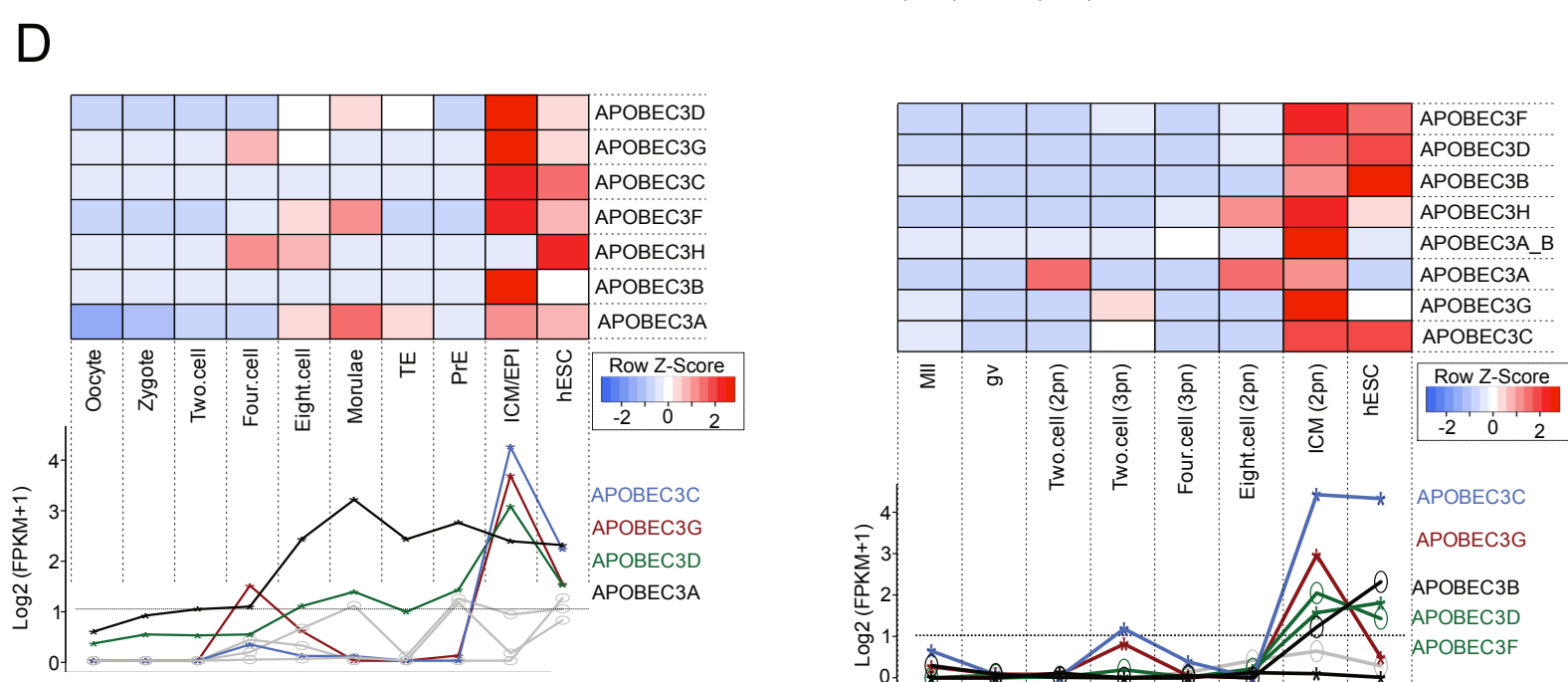

Supplement: S8 Fig — Code to generate these figures is at doi.org/10.5281/zenodo.7925199. (A) Line plot showing the expression of APOBEC3G in ICM, KD-GFP (Control) (2 replicates), HERVH [35] (2 replicates) in hESC_H1 and TE. Note that the relative expression of APOBEC3G is the highest in ICM, depleted in KD-HERVH and the lowest in TrEs. (B) Boxplot represents the distribution of relative average difference (at Log2 scale) of gene expression around HERVH loci. Single cells are pooled together, scaled, and averaged for the analysis. Only genes, neighbouring HERVH (10 KB window) and expressed at least in 10% of the cells are used for the analysis. Note: Up-regulated gene expression neighbouring HERVH (HERVH target) was specifically observed in ICM but neither in NCC nor in pre-TE transcriptomes. (C) Representative examples of chromatin loops (boxed, red-HERVH; blue-APOBECs) between HERVH and the APOBEC3 genes in hESCs (zoom in, 5 KB resolution). Heatmaps show normalised counts of Hi-C reads between selected genomic loci pairs. Gene structure, phylogenetic conservation, SINE, LINE, LTR retroelements, and 3D interaction at the APOBEC3 (chr22) locus are shown. (D) Heatmap showing the scaled expression (Log2 FPKM) dynamics of the APOBEC3 family of genes during the preimplantation life of human embryo (left panel [44], right panel [1]). Blue denotes lower to no expression. Line plot beneath the heatmap shows the averaged raw expression (Log2 FPKM+1) of the APOBEC3 genes. (PDF) [file pbio.3002162.s008.pdf]

A

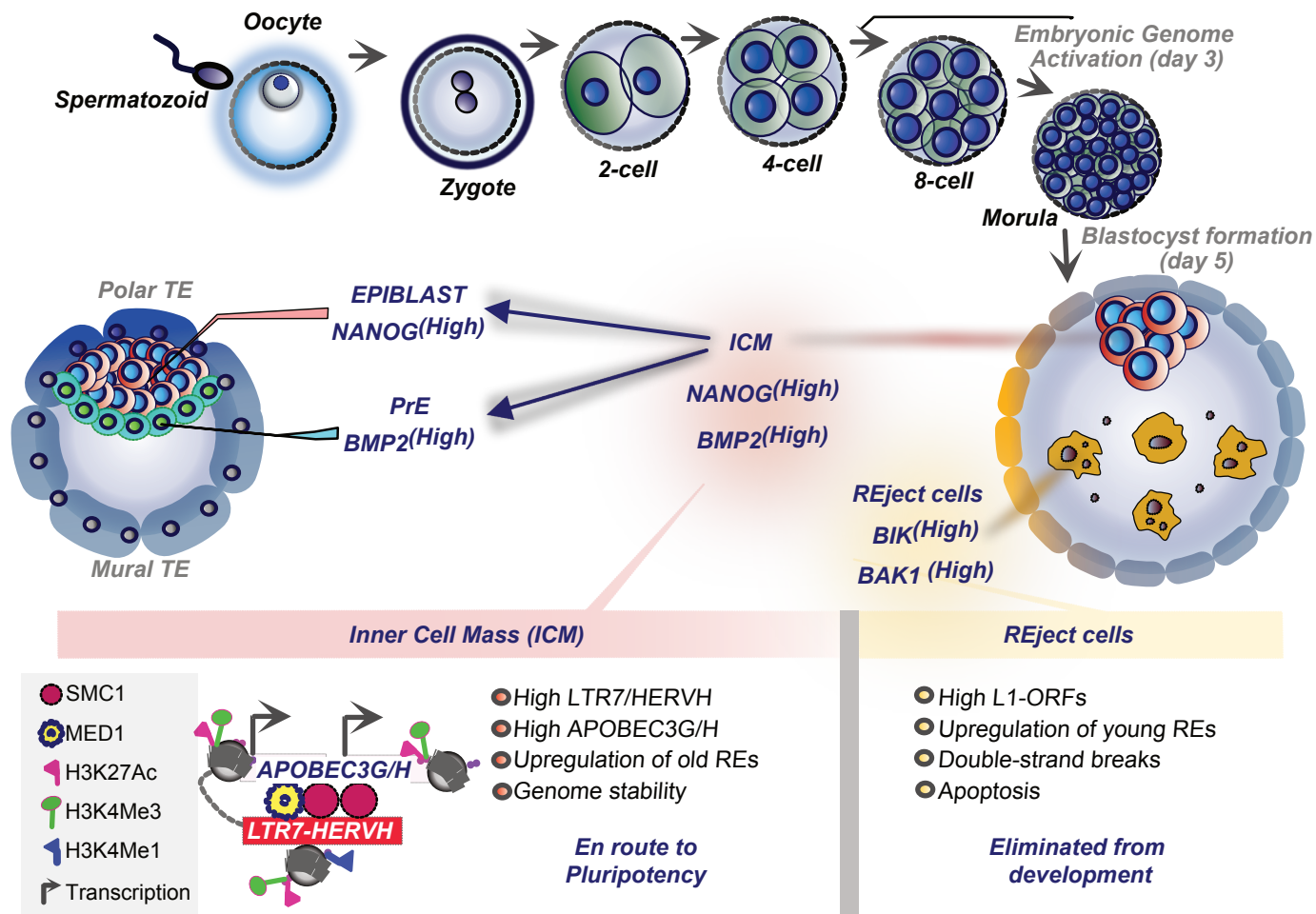

B

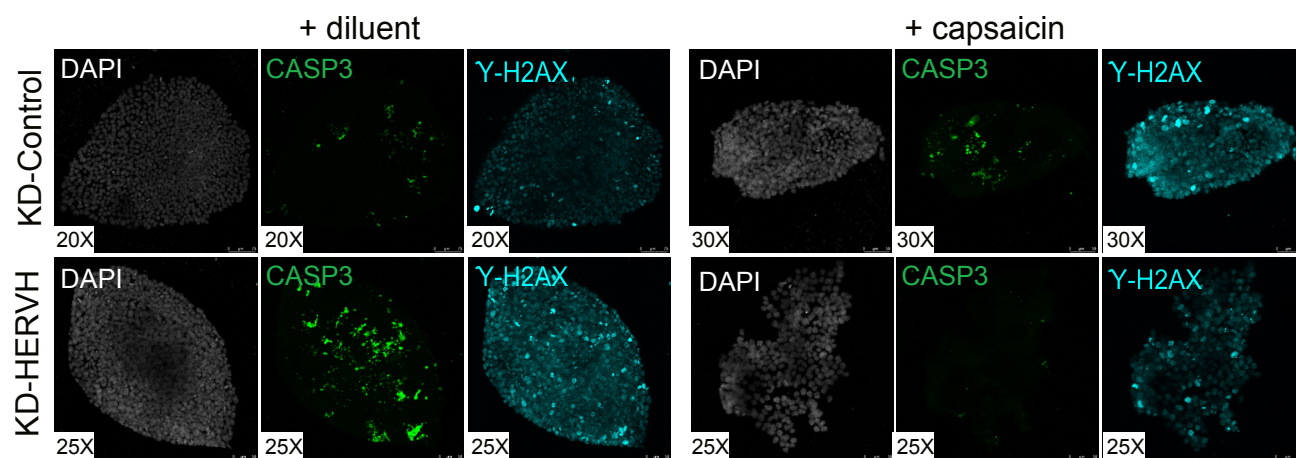

C

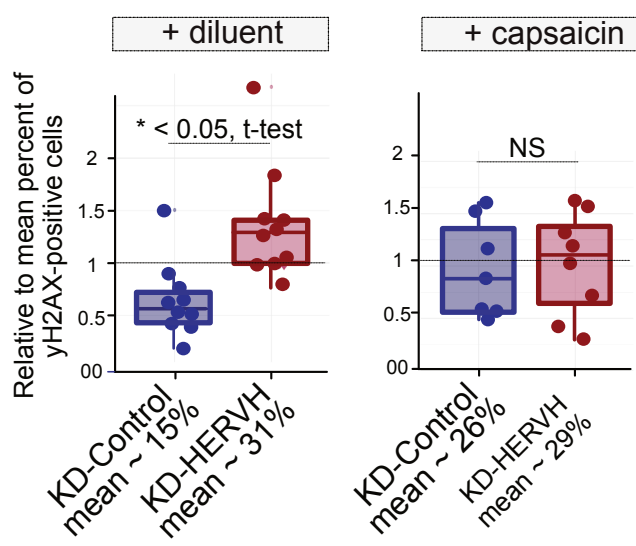

D

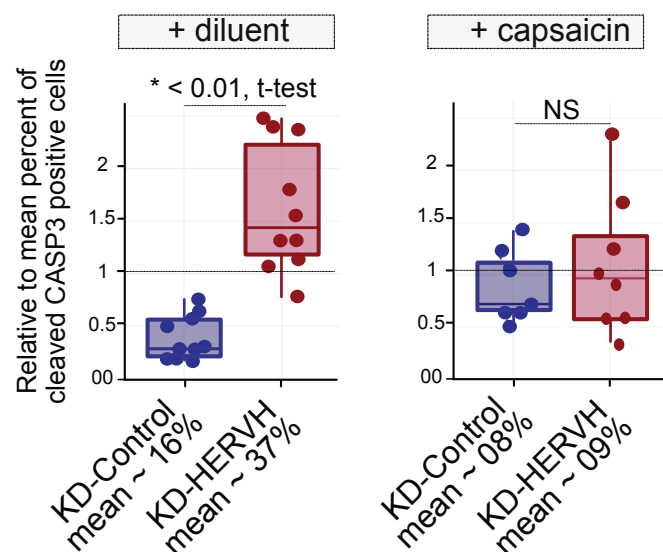

Supplement: S9 Fig — Code to generate these figures is at doi.org/10.5281/zenodo.7925199. (A) The selection arena model in early human embryos. (B) Capsaicin treatment antagonises the effect of HERVH suppression in hESCs. HERVH suppression (KD) results in increased DNA damage and apoptosis in hESCs (visualised by γ-H2AX and cleaved cl_CASP3 immunostaining, respectively), but mitigated by capsaicin treatment. KD-HERVH_H9 and KD-Control_H9 (scramble). (C) Knocking down HERVH results in elevated DNA damage in KD-hESC_H9 (visualised by γ-H2AX immunostaining (n = 3)). However, inhibition of L1 activity by capsaicin reduces the DNA damage, visualised by immunofluorescent staining against γ-H2AX in KD-hESC_H9. KD-Control (scrambled). Error bar–standard error of the mean. Diluent, ethanol (n = 3). (D) Knocking down HERVH results in an enhanced apoptotic signal (cl_Caspase3). However, inhibition of L1 activity by capsaicin reduces the pre-apoptotic signal in KD-hESC_H9. Diluent, ethanol. KD-Control (scrambled). Error bar–standard error of the mean. (PDF) [file pbio.3002162.s009.pdf]

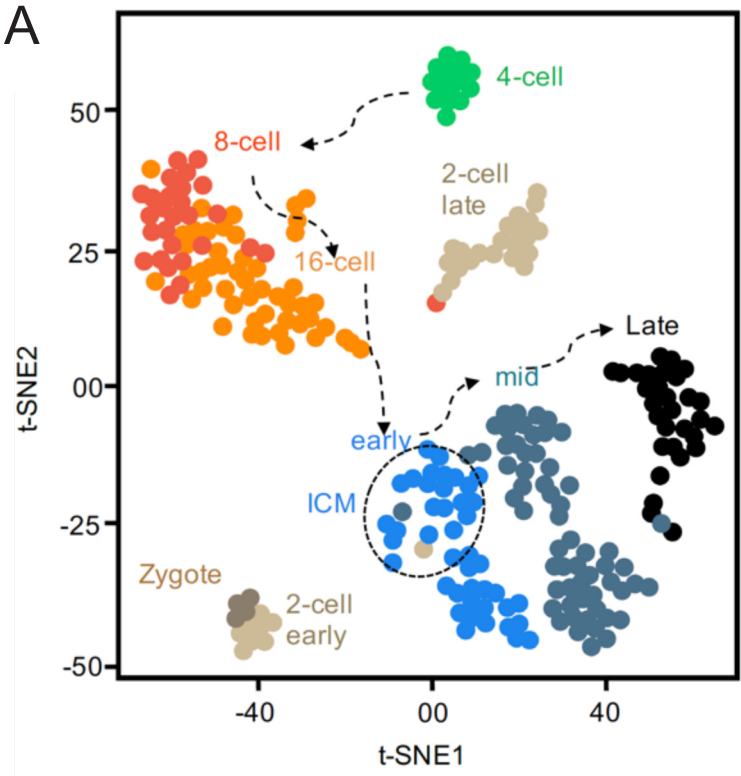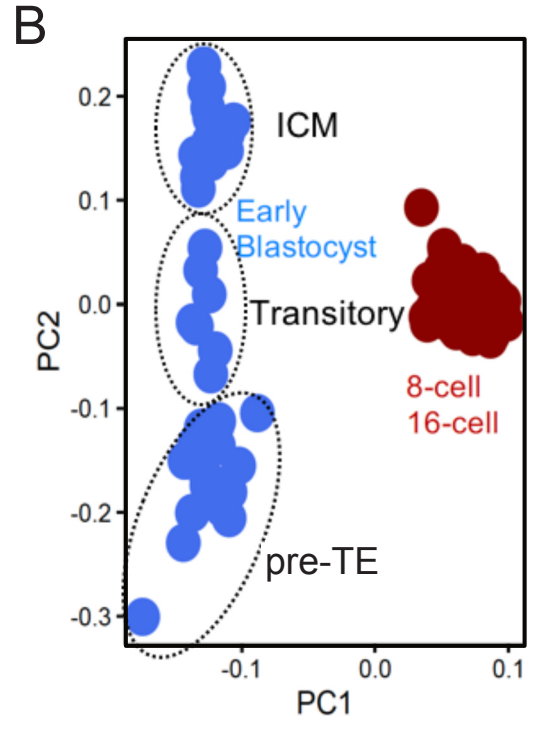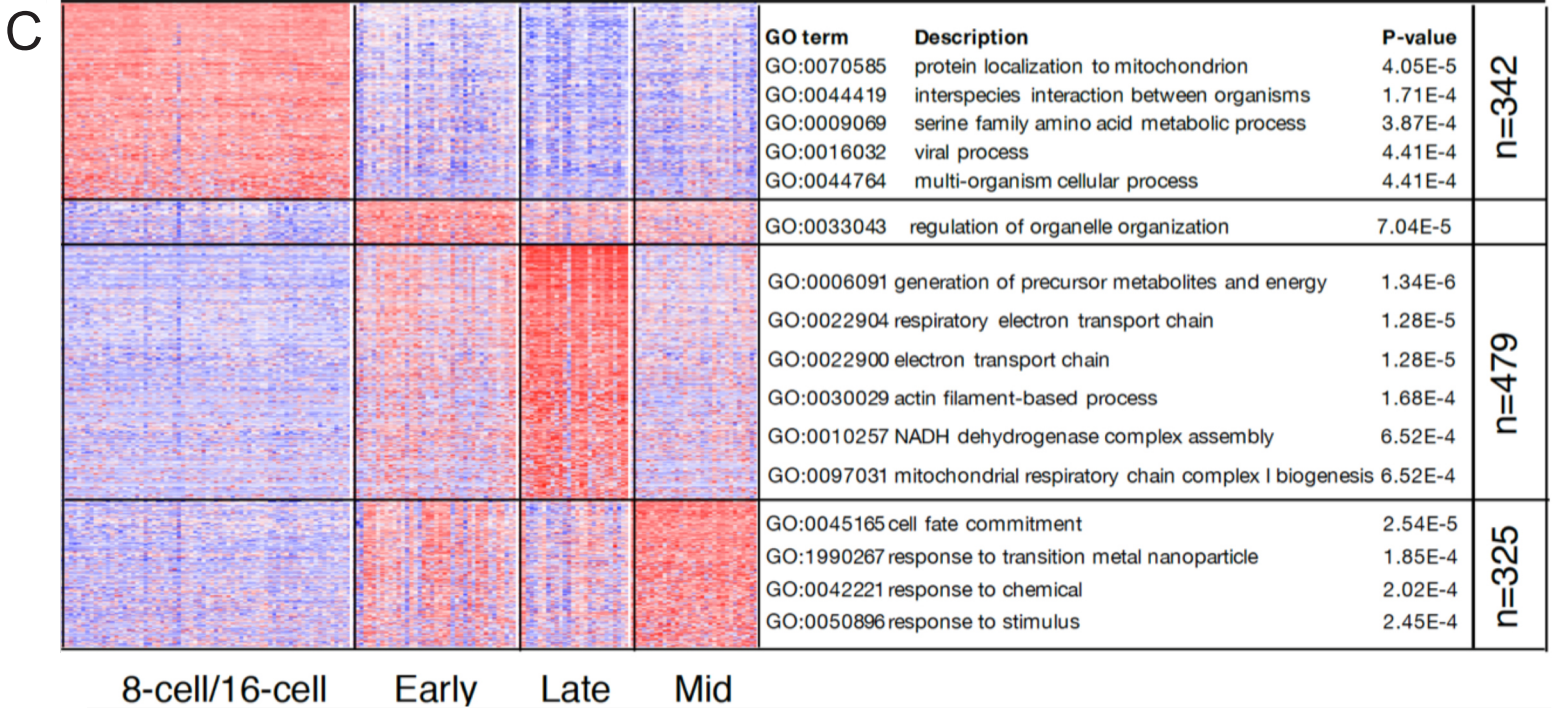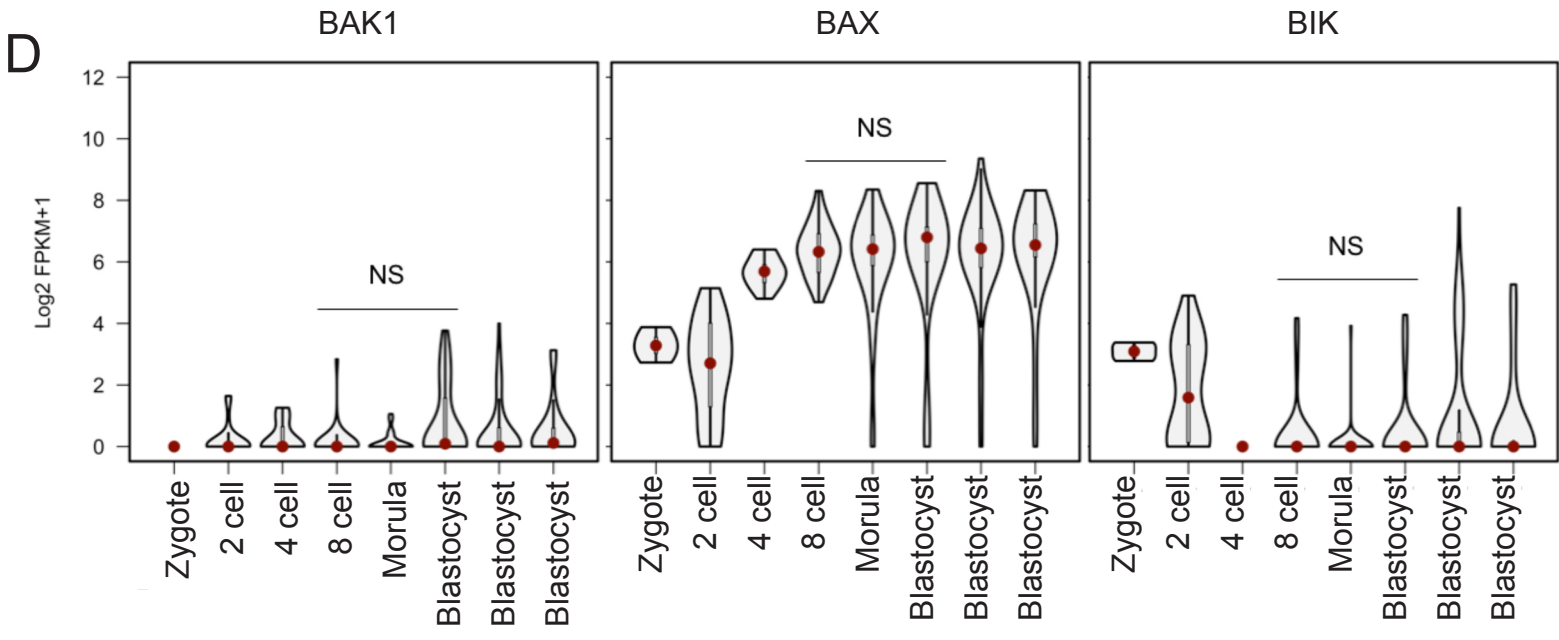

Supplement: S10 Fig — Code to generate these figures is at doi.org/10.5281/zenodo.7925199. (A) Two-dimensional tSNE density cluster representation of 259 mouse single-cell preimplantation transcriptomes [99] using the 1,297 MVGs (analysed similarly to the human data). Dashed, curvy arrows indicate the progression of the embryogenesis. (B) PCA of morula and early blastocyst stages. The segregated clusters are based on the expression of MVGs. ICM (marked by NANOG), pre-TE (marked by CDX2), and transitory state (marked by NANOG/CDX2) are circled. (C) Heatmap displaying z-scores of up-regulated genes (red) during mouse preimplantation embryogenesis. Gene Ontology of genes enriched 2-fold in a particular developmental stage compared to the rest of the developmental stages. The data frame uses normalised mean TPM (calculated per mouse embryonic stage) 8-cell, morula and early/mid/late blastocysts. (D) Violin plot visualisation of the expression dynamic of selected pro-apoptotic marker genes in mouse embryogenesis. Note: There is no significant difference in pro-apoptotic marker gene expression following morula stage (NS, not significant). (PDF) [file pbio.3002162.s010.pdf]
