# Supplementary material for: A new human embryonic cell type associated with activity of young transposable elements allows definition of the inner cell mass
Source: PLoS Biol. 2023 Jun 20;21(6):e3002162. doi: 10.1371/journal.pbio.3002162 (PMC10281584; doi:10.1371/journal.pbio.3002162)
Supplement: Attachment — Submitted filename: PlosB_Response_reviewers_08032023.pdf [file pbio.3002162.s017.pdf]

Reviewer #1

In this manuscript, Singh et al, reanalyze published scRNAseq of human preimplantation embryos to distinguish ICM cells from their cells states at 5 d.p.f. This med them to identify pre-apoptotic cells. They then focus on ICM cells and study the activity of transposable elements. This raises an interesting discussion on how embryos are protected from transposable elements, relatively to their "age".

While of potential interest, this article suffers from a major narrative issue (as noted by previous reviewers). More efforts are needed to answer those previous comments.

Response: We are unclear what the referee is referring to here. None of the points below relates to narrative issues raised previously. More particularly, the significant narrative issue in prior versions has been addressed. This is the cause-and-effect issue, now in the discussion praised by referee 2 who notes: *"One major highlight of the present manuscript is the detailed and insightful discussion, using the data generated for this study and a very comprehensive review of the literature, to begin to explain the mechanism by which unwanted/aneuploid cells may be diverted from the emerging epiblast".*

Find below points that could help improve the manuscript.

We thank the Reviewer for their suggestions.

Major:

1/ I understand that this article might have been around for a long time, but it needs to be rewritten considering the state of the art at the moment it will be published, otherwise it will look odd. Maybe start by saying you are performing a complementary approach to Radley et al in order to define human ICM and clearly identify the "other" cells that are generally ignored.

Response: The reviewer is asking for helpful textual changes. The two new key papers are those of Radley et al., and Meistermann et al., for the ICM identification and two-step model of blastocyst lineage specifications, respectively. As we don't approach the

latter issue until the discussion, the main paper requiring special contextual appraisal is that of Radley et al.

We are fortunate that Radley cite our early version of the present paper thereby making the contextual issues easier to address. They summarise their findings by proposing SPIC and PRSS3 as ICM markers then cite our paper stating, "*We note that previous studies have proposed SPIC and PRSS3 as human ICM markers (Singh et al., 2019)*". Given that Radley et al. refer to our work, we believe it would be unfair to both papers to say that our work simply starts by performing a complementary approach to Radley et al. to define human ICM (this would also be a somewhat inaccurate summary of the state of acknowledged precedence).

Nonetheless, we do not wish to take anything away from Radley et al.. The ICM finding using the complex algorithms in their paper is an important contribution and must be cited in the present manuscript. We have duly added both references to the manuscript stating: *We note that Radley et al. [4], using a state-of-art set of algorithms, recently acknowledged that they could confirm the same marker genes as we report (presented in the early release of the present paper [14]).* We also cite them early on as an analysis on single cell data pulling apart various cell types.

The relationship with Meistermann et al. is more straightforward as to a large extent we don't overlap greatly. In the introduction we note that Meistermann didn't identify the ICM but did identify a novel cell population (progenitors to PE). We have substantially changed the introduction to better reflect this and the state of the art more generally.

In addition, in the discussion we state:
"This rejects the hypothesis of simultaneous blastocyst lineage specification in human early embryos [2]. Since we first reported this [14], the finding has been replicated in recent studies, via diverse methods [3, 40]."

We hope that referee (and the journal) agrees that our changes allow both correct allocation of precedence while also recognizing the current context.

We hope that the intent of the paper is also now clearer and is "*to define the cell types of the early human embryo more fully*, *with a particular focus on the activity of TEs".*

For clarity, as regards defining "other" cells, we classify every cell from Petropolous data (Fig 1A). We now also remark on the E6 cell type expressing TE, EPI and PE markers, mostly the PE markers. We note that these may well be comparable to the cell population recently discovered by Meistermann et al. Our analysis likewise clarifies what Meistermann, called "other" cells: from E5, E6, and E6-7 stages. As we report, the E6-7 belonged to Mural TE, and we know the identity of all E5 cells. The ones uncharacterized before were either NCC or transitionary cells.

2/ The name "NCC" does not make sense if those cells are undergoing apoptosis sooner or later. Instead, I would insist on the fact that an important % of cells in human embryos have failed specification and are dying (you could speculate about their potential aneuploidy in the discussion). This is supported by the nice deconvolved bulk RNAseq, and it is novel. Authors could show was the annotation of the "other" cells in Radley et al or Meistermann et al, for instance, to show that it is important to identify those cells in order to clean up subsequent analyses.

Response: If we have understood correctly, we appreciate the problem the referee raises: non-committed could be read as "yet to commit". If so, we agree that the language could be confusing. Despite this, after reflection, we have opted to retain the original non-committed cells (NCC) title. Our central concern is to name them after their defining feature when first identified rather than their inferred future trajectory. As our definition relies on the absence of commitment markers at E5 this should, we think, be reflected in their naming. Importantly, this also means that we do not need to presume that all NCC cells share the same fate. We have shown that many do progress to apoptosis. However, showing that 100% of NCCs progress to this fate is not a claim we would be comfortable making with the current data. A term such as "pre-apoptotic" for example would imply no functional heterogeneity within the NCC population. More prosaically, we would be skewing the flow of the manuscript narration by prejudging their biology. While then the possibility of misinterpretation remains, anyone reading the paper in full should not be confused what NCCs are nor their likely fate. We hope the referee can respect our choice.

We have added a very brief defense to the manuscript: "*Note that this naming makes no presumptions about homogeneity of fate of these cells and reflects instead their definitional characteristics.*"

As regards classification of "other" cells, we do classify all cell types (see above).

3/ The abstract/introduction suffer from erroneous statements, truly impairing credibility of the work. For instance:"On a single-cell level, there are uncharacterised cell types": what does that mean?

Response: The statement was really rather modest and we aren't sure why the reviewer considers the statements so egregious: we just meant that some cell types weren't annotated. In Petropolous's data (ArrayExpress: E-MTAB-3929) for example at E5 most cells are classified as either trophoectoderm, epiblast or PE, but numerous exactly contemporaneous cells have no indicative annotation. We struggle to see why noticing this truly impairs the credibility of our work. Perhaps it would have been clearer if we said unannotated? Regardless, we delete the relevant sentences as they are unnecessary to motivate the paper.

"Perhaps most importantly, we still have no clear definition of the inner cell mass (ICM), the transitory group of pluripotent cells from which embryos are derived and hence of interest in regenerative medicine" ICM are the progenitors of EPI which are pluripotent. Embryos also refers to the placenta, you could use foetus, alternatively. Main application of human preimplantation development is IVF.

Response: We have now changed this sentence: "*Perhaps most importantly, the inner cell mass (ICM), from which the foetus is derived and hence of interest in reproductive health and regenerative medicine, has proven hard to define.*"

4/ The quality of IFs in fig2 is really sub-standard.

Response: We have now enhanced the resolution of all immunostaining results presented in Figure 2. The resolution of our figure now provides clear visibility of stained single nuclei (and its resolution accords with the journal's standards).

5/ Fig 1A: the "mural" and "polar" cluster are odd (really small) compared to previous studies such as Meisterman et al. The big E6-E7 cluster looks like mural TE.

Response: This is an excellent suggestion. Upon closer inspection, we, too, find that the big E6-E7 cluster is indeed mural TrE. This was also evident from the TrE cells with upregulation of DLX3 and downregulation of CYP19A1 (Polar TrE marker) expression shown in Supplementary figure 1C. So, following the reviewer's instruction, we annotate the big E6-E7 cluster as "Mural TrE). We have now modified figure 1A (and the legend).

Minor:

1/ it is better to use d.p.f. rather than "E" for human embryos.

Response: We commenced our study by analyzing the data published of Petropolous et al and Yan et al., who use "E" for embryonic stages. We would like to be consistent with that terminology so the reader can see the relationship between what we present and their data. We, however, acknowledge the reviewer's view and add a sentence that our "E" terminology corresponds to the d.p.f. (day post fertilization) (Fig 1a legend).

2/ what is the RNA velocity of "NCC"

Response: The reviewer is referring to Line number 32, where the sentence states, "The high diffusion distance, and the lack of directionality from RNA velocity of NCCs, indicate that these cells do not follow the developmental trajectory of either ICM or pre-TrE after embryonic gene activation (EGA) through the progression of blastocyst formation (Fig 1B-C and S2A-D Fig)"

For clarity we modify the sentence to: "*The high diffusion distance and the lack of directionality from RNA velocity analysis of these three E5 lineages indicate that NCC does not follow the developmental trajectory of either ICM or pre-TrE after embryonic gene activation (EGA) through the progression of blastocyst formation (Fig 1B-C and S2A-D Fig)*".

3/ you seem to have inverted TE (Trophectoderm) and TrE (Transposable elements)?

Response: Thank you for spotting this. We have found three instances where TE abbreviation was inverted. These are all corrected in the revised version.

4/ your yellow-violet scale in heatmaps is inverted: the warm color (yellow) should be for high expression.

Response: There are three heatmaps in this manuscript with the colour scales pointed out by the reviewer. We modified all three heatmaps to a more suitable colour scale, one used in Meistermann et al., 2021 (displayed in Figure 1E, Supplementary Figure S2E, and Supplementary Figure S8D).

Reviewer #2

In this manuscript, Singh and co-workers seek to scrutinise the identity and fate of cells within the human inner cell mass, exploring the role of transposable elements in particular. The authors search published single cell multi-omics data obtained from human and other primate embryos, perform immunofluorescence and make use of human embryonic stem cells as an *in vitro* model. They uncover populations of cells associated with cell death and others appearing uncommitted, which they call 'non-committed cells (NCCs)'. They use published data to characterise pre-epiblast stage cells, revealing that NCCs are represented only up until ~E5, and tend to express pro-apoptotic genes. To control against the risk that handling for single cell isolation causes cell death they processed some morulae and ICMs by bulk sequencing and found the same proportion of dead cells as encountered using separated cells, which is an important, but often ignored validation. Further analysis of NCCs demonstrated that they express BCL2-Interacting Killer (BIK) and other genes that foretell programmed cell death. They also show markers of DNA damage. By immunofluorescence the authors found mutual exclusivity for NANOG and the death mark, gamma H2AX, implying that markers of apoptosis are not part of the ICM repertoire, as previously thought (and previously reported in the ICMs of developing mouse embryos), but transitory and excluded after E5. Early (E5) ICMs exhibit 3 clusters: Trophoblast, EPI/PE precursors and NCCs. To monitor how the lineages resolve during ICM maturation, embryos were stained for NANOG and BMP2. Several 'ultra-hot' Line 1 ORFs encoding ORF1P elements are observed in E5-6 embryos, but do not overlap with OCT4, therefore assigned TrE identity, also consistent with position within the embryo. This population of TrE cells co-stains with Caspase 3, so probably represents cells displaced to the extra-embryonic tissues, as previously suggested. ICM and NCC have distinct families of TEs (Old versus Young, respectively). One of the Old ones (HERVH-int) is considered to be a binding site for pluripotency transcription factors. The authors create a model for the pre-differentiating ICM using hESCs. High HERVH cells (marked with GFP expression tag) correspond with ICM and EPI pluripotency markers. TE-restricting factors tend to be exclusively in ICM. The 'Young' TEs are suppressed by APOBEC3, activated by LTR7/HERVH in ICM.

The data presented in this study provide further evidence for sequential rather that synchronous divergence of the founder lineages of the blastocyst, which was first published in a massive, high profile single cell sequencing study, but subsequently refuted using more refined analysis. One major highlight of the present manuscript is the detailed and insightful discussion, using the data generated for this study and a very comprehensive review of the literature, to begin to explain the mechanism by which unwanted/aneuploid cells may be diverted from the emerging epiblast.
Finally, the authors propose that TEs may regulate transition between developmental stages in human as well as other mammals.

The authors have made every effort to address comments from reviewers when this work was under consideration with a different publisher, strengthening the manuscript considerably and I have nothing further to criticise.

Response: We thank the reviewer for the kind words and positive assessment.
