# Supplementary material for: A new human embryonic cell type associated with activity of young transposable elements allows definition of the inner cell mass
Source: PLoS Biol. 2023 Jun 20;21(6):e3002162. doi: 10.1371/journal.pbio.3002162 (PMC10281584; doi:10.1371/journal.pbio.3002162)
Supplement: Attachment — Submitted filename: Nichols_PLOSB.docx [file pbio.3002162.s016.docx]

In this manuscript, Singh and co-workers seek to scrutinise the identity and fate of cells within the human inner cell mass, exploring the role of transposable elements in particular. The authors search published single cell multi-omics data obtained from human and other primate embryos, perform immunofluorescence and make use of human embryonic stem cells as an *in vitro* model. They uncover populations of cells associated with cell death and others appearing uncommitted, which they call ‘non-committed cells (NCCs)’. They use published data to characterise pre-epiblast stage cells, revealing that NCCs are represented only up until ~E5, and tend to express pro-apoptotic genes. To control against the risk that handling for single cell isolation causes cell death they processed some morulae and ICMs by bulk sequencing and found the same proportion of dead cells as encountered using separated cells, which is an important, but often ignored validation. Further analysis of NCCs demonstrated that they express BCL2-Interacting Killer (BIK) and other genes that foretell programmed cell death. They also show markers of DNA damage. By immunofluorescence the authors found mutual exclusivity for NANOG and the death mark, gamma H2AX, implying that markers of apoptosis are not part of the ICM repertoire, as previously thought (and previously reported in the ICMs of developing mouse embryos), but transitory and excluded after E5. Early (E5) ICMs exhibit 3 clusters: Trophoblast, EPI/PE precursors and NCCs. To monitor how the lineages resolve during ICM maturation, embryos were stained for NANOG and BMP2. Several ‘ultra-hot’ Line 1 ORFs encoding ORF1P elements are observed in E5-6 embryos, but do not overlap with OCT4, therefore assigned TrE identity, also consistent with position within the embryo. This population of TrE cells co-stains with Caspase 3, so probably represents cells displaced to the extra-embryonic tissues, as previously suggested. ICM and NCC have distinct families of TEs (Old versus Young, respectively). One of the Old ones (HERVH-int) is considered to be a binding site for pluripotency transcription factors. The authors create a model for the pre-differentiating ICM using hESCs. High HERVH cells (marked with GFP expression tag) correspond with ICM and EPI pluripotency markers. TE-restricting factors tend to be exclusively in ICM. The ‘Young’ TEs are suppressed by APOBEC3, activated by LTR7/HERVH in ICM.

The data presented in this study provide further evidence for sequential rather that synchronous divergence of the founder lineages of the blastocyst, which was first published in a massive, high profile single cell sequencing study, but subsequently refuted using more refined analysis. One major highlight of the present manuscript is the detailed and insightful discussion, using the data generated for this study and a very comprehensive review of the literature, to begin to explain the mechanism by which unwanted/aneuploid cells may be diverted from the emerging epiblast.

Finally, the authors propose that TEs may regulate transition between developmental stages in human as well as other mammals.

The authors have made every effort to address comments from reviewers when this work was under consideration with a different publisher, strengthening the manuscript considerably and I have nothing further to criticise.
