# Supplementary material for: A new human embryonic cell type associated with activity of young transposable elements allows definition of the inner cell mass
Source: PLoS Biol. 2023 Jun 20;21(6):e3002162. doi: 10.1371/journal.pbio.3002162 (PMC10281584; doi:10.1371/journal.pbio.3002162)
Supplement: Attachment — Submitted filename: Response to reviewers.docx [file pbio.3002162.s018.docx]

Response to reviewers/editors

Regarding the comments:

1. We adopt the title suggested by you.
2. Trophectoderm we abbreviate to TE and transposable elements, as retroelements, we abbreviate RE. We also explain:

“While about half of our genome consists of remnants of transposable elements [9], transposition of some Young retroelements (REs) (< 7 MY, human-chimpanzee split), mobilized via retrotransposition, is possible [10, 11]”

3. This resolves what pre-TE refers to.
4. We abbreviate primitive endoderm as PrE.
5. In the manuscript we first (temporarily) define cells as “Not characterised” cells = NCCs. Having characterised them we then note that as all the transposable elements are Retro Elements and the cells are discarded from the developmental process, we dub the new cells as REjects. We thus avoid usage of the term “non-committed”).
6. There was one issue that we don’t fully understand, not least because there seems some confusion. You write “*Regarding the naive issue, it seems that the paper contains experiments on primed cells and also a demonstration in the supplement figures with naive. Thus, this must be discussed to consider why primed cells should have this population, and the major data is from primed ESCs*.”

First, to clarify, we don’t employ “classical” naïve cells. We employ primed cells and HERVH^high^ (alias GFP^high^) cells that are referred as “naive-like” [doi: 10.1038/nature13804]. However, these would not be classified as naïve by most others in the field as their “naïve” cells do not have HERVH high. Of note, these naïve-like cells have a higher accessible chromatin activity, but just not the higher level of HERVH RNA from the polyA transcriptomic repertoire. “Classical” naïve cells express some Young REs, and are thought to capture some features of the morula-early blastocyst stage (doi: 10.1016/j.stem.2016.06.011). In contrast, HERVH^High^ naïve-like cells, with low Young RE expression resemble a somewhat later stage of embryogenesis (more ICM-like). Additionally, we show in Figure 4B that the ICM and epiblast markers discovered in current and previous studies (Meistermann et al., and Radley et al.) are specifically expressed and are thus relatively upregulated in HERVH^High^ cells.

We aren’t quite sure what you mean by “*have this population*”. What is *this*? We think you mean, why does a population of primed cells have NCC cells in their midst? If so, this is also a misunderstanding. We also look in the same hESC population for HERVH^low^ cells (i.e with medium/lower levels of HERVH expression) to see what differs between low and high. Because HERVH^high^ cells comprise only ~5% of hESCs [doi: 10.1038/nature13804], these HERVH low cells are classical primed cells, not NCC like cells. This comparison shows what happens when HERVH is increased. In addition, we compared hESCs to HERVH-KD. This shows what happens when HERVH is made to be depleted. Both reveal activity on the same axes: HERVH controls genome stability and Young transposable elements. But note, we do not claim that HERVH^low^ cells are at all like the new cell type. Our new cell type is not naturally found in ESC populations, but we can manipulate similar cells to see what happens when HERVH is drastically reduced (HERVH-KD). We have amended the text to avoid any possible confusions:

“Note that the KD cells are not the same as HERVH^low^ cells, the latter being primed hESCs and there are no mimics of the NCC cells in ESC populations. Nonetheless, cells can be generated that capture some features of NCCs (RE upregulation) by knocking down HERVH in ESCs.”

What hope that this addresses any confusions.

7. We clarify the knowns and unknowns of ICM definition. Specifically, we write:

“Perhaps most importantly, the key cell type from which the embryo is derived, the ICM, has for long while remained less well resolved in both its ontogeny and its definition [2, 5][see also 3]. Indeed, for a considerable period, ICM was thought too transitory to capture and identify [2, 5]. The definition is, however, a key goal, not least because it would enable a better understanding of human pluripotency and the comparative nature of laboratory model cells, human embryonic stem cells (hESCs).

While expression of certain pluripotency genes and transcription factors all but define ICM (e.g. NANOG, GATA6), recent further characterization additionally included apoptotic genes (e.g. in [6]), which is perhaps unexpected.”
